# Development and anticancer properties of Up284, a spirocyclic candidate ADRM1/RPN13 inhibitor

**DOI:** 10.1371/journal.pone.0285221

**Published:** 2023-06-14

**Authors:** Ravi K. Anchoori, Vidyasagar Anchoori, Brandon Lam, Ssu-Hsueh Tseng, Samarjit Das, Fernanda Carrizo Velasquez, Balasubramanyam Karanam, Deepika Poddatoori, Ramesh Patnam, Michelle A. Rudek, Yung-Nien Chang, Richard B. S. Roden

**Affiliations:** 1 Department of Oncology, Johns Hopkins University, Baltimore, MD, United States of America; 2 Department of Pathology, Johns Hopkins University, Baltimore, MD, United States of America; 3 Up Therapeutics LLC, Frederick, MD, United States of America; 4 SV Chem Biotech, Edmonton, AB, Canada; 5 Department of Anesthesiology and Critical Care Medicine, The Johns Hopkins University, Baltimore, Maryland, United States of America; 6 Department of Biology and Center for Cancer Research, Tuskegee University, Tuskegee, Alabama, United States of America; 7 Prochem Organics, IDA Pashamylaram, Isnapur, Medak, Telangana, India; 8 Department of Medicine, Johns Hopkins University, Baltimore, MD, United States of America; B. S. Abdur Rahman Crescent Institute of Science and Technology, INDIA

## Abstract

Bortezomib has been successful for treatment of multiple myeloma, but not against solid tumors, and toxicities of neuropathy, thrombocytopenia and the emergence of resistance have triggered efforts to find alternative proteasome inhibitors. Bis-benzylidine piperidones such as RA190 covalently bind ADRM1/RPN13, a ubiquitin receptor that supports recognition of polyubiquitinated substrates of the proteasome and their subsequent deububiqutination and degradation. While these candidate RPN13 inhibitors (iRPN13) show promising anticancer activity in mouse models of cancer, they have suboptimal drug-like properties. Here we describe Up284, a novel candidate iRPN13 possessing a central spiro-carbon ring in place of RA190’s problematic piperidone core. Cell lines derived from diverse cancer types (ovarian, triple negative breast, colon, cervical and prostate cancers, multiple myeloma and glioblastoma) were sensitive to Up284, including several lines resistant to bortezomib or cisplatin. Up284 and cisplatin showed synergistic cytotoxicity *in vitro*. Up284-induced cytotoxicity was associated with mitochondrial dysfunction, elevated levels of reactive oxygen species, accumulation of very high molecular weight polyubiquitinated protein aggregates, an unfolded protein response and the early onset of apoptosis. Up284 and RA190, but not bortezomib, enhanced antigen presentation *in vitro*. Up284 cleared from plasma in a few hours and accumulated in major organs by 24 h. A single dose of Up284, when administered to mice intra peritoneally or orally, inhibited proteasome function in both muscle and tumor for >48 h. Up284 was well tolerated by mice in repeat dose studies. Up284 demonstrated therapeutic activity in xenograft, syngeneic and genetically-engineered murine models of ovarian cancer.

## Introduction

Targeted protein modification using small molecule covalent inhibitors is a validated approach for the treatment of cancers (e.g. carfilzomib, ibrutinib, afatinib and osimeritinib), and they appear less prone to resistance [1]. Addressing the emergence of drug resistance is a key challenge in cancer therapy, for example patients with high grade ovarian cancer who relapse at high rates despite aggressive debulking surgery and first line platinum-based chemotherapy. Michael acceptors are covalent inhibitors that act by forming covalent bonds with cysteines or other nucleophilic residues in the target protein. Several Michael acceptor-based inhibitors targeting the proteasome’s 19S Regulatory Particle (19S RP) have potential for cancer treatment, and VLX1570 advanced to clinical testing [2–4]. However, many are as Pan-Assay interference compounds (PAINS) which tend to react non-specifically. A number of *in silico* filters are used in drug design to screen out PAINS [5, 6].

Several proteasome inhibitors (bortezomib, carfilzomib, and ixazomib), targeting its 20S catalytic particle (20S CP) are licensed for the treatment of multiple myeloma (MM) [7, 8]. These peptide-based drugs have greatly improved the prognosis of MM patients, but in early phase trials these drugs were ineffective against solid tumors [9]. Additionally, prolonged treatment of MM patients is associated with off-target neurotoxicity, thrombocytopenia and eventually resistance [8, 10]. This suggests a need for a new class of proteasome inhibitors with a distinct mechanism of action and molecular scaffold to overcome these limitations.

An emerging strategy is to target substrate recognition and deubiquitination mediated by the 19S RP as it selects polyubiquitinated proteins as substrates and prepares them for proteasomal degradation via deubiquitination and unfolding [11]. The 19S RP is pivotal for constitutive 26S proteasome activity and its inhibition causes a rapid and toxic buildup of its substrate polyubiquitinated proteins in very high molecular weight aggregates. Ovarian cancer cells, like MM, produce dramatic amounts of misfolded proteins because of their aberrant metabolism [12]. Consequently, these cancer cells heavily rely on proteasomes to maintain protein homeostasis, thereby creating a therapeutic window [13, 14]. Proteasomes are also used to modulate the levels of regulatory proteins and thereby control complex cellular processes such as cell cycle and DNA repair that are critical to tumor growth and resistance to platinum-based chemotherapy.

The 19S RP subunit protein RPN13, which is encoded by *ADRM1*, has emerged as a promising target for the treatment of ovarian cancer and several other types of solid and liquid tumors [15–21]. RPN13 was found non-essential for the survival of normal cells but is critical in numerous cancer cell lines, including cells from solid tumors [15, 22, 23]. Indeed, *ADRM1* has been proposed as a driver oncogene in ovarian cancer [21]. RPN13 works in concert with RPN10 to recognize with high affinity the extended K48-linked polyubiquitinated chains marking proteins for proteasomal degradation [11]. UCH37, a proteasomal deubiquitinase (DUB), binds to and is activated by RPN13 [24]. Deubiquitination is a prerequisite for further substrate unfolding and degradation by proteasome. Thus, inhibition of RPN13 has the potential block all proteolytic activity of the constitutive 26S proteasome by preventing substrate recognition and deubiquitination, whereas the three licensed proteasome inhibitors only target one of the three proteolytic activities (chymotryptic) encoded by its 20S catalytic core, limiting their potency and potentially providing a window for resistance to emerge [11].

RA190 was the first putative small molecule RPN13 inhibitor (iRPN13) [19]. RA190 inhibits tumor growth in ovarian, cervical, and MM xenograft models. Intriguingly, it retains potency in bortezomib-resistant MM cells. However, RA190 and its more potent analogs have suboptimal characteristics for a drug, including PAINS characteristics because of its central piperidone core. Chang described novel iRPN13 possessing instead a central spiro-carbon ring that does not fall within the PAINS category [5, 6, 25]. The spiro-scaffold offers ring strain and limits number of stable confirmations compared to a cyclohexane moiety, but a very strong withdrawing group, such as–NO_2_, on the aromatic ring is required for cytotoxicity against tumor cells. The cytotoxicity of 3,4 and 3,5 spirocyclic compounds bearing–NO_2_ functional groups was similar, but due to better access to raw materials and synthetic possibilities, the 3,5 spirocyclic compounds were further developed (US20190365726). The 3,5 spirocyclic compound Up109 emerged as a promising candidate that bound to RPN13 *in vitro* more potently than RA190 and caused accumulation of high molecular weight (HMW) polyubiquitinated (polyUb) proteins, unresolved endoplasmic reticulum (ER) stress, and apoptosis. Furthermore, Up109 treatment inhibited *in vivo* proteasome function and growth of ovarian cancer xenograft ES2 (US20190365726). However, issues with the solubility of Up109 and toxicity necessitated further development of a more drug-like iRPN13.

Here we describe the development of a spirocyclic iRPN13, Up284. Up284 shows promising safety, pharmacokinetics and therapeutic activity in several murine models of ovarian cancer. Up284 also synergizes with the platinum-based chemotherapeutic cisplatin that acts by inducing DNA damage. DNA damage repair mechanisms are compromised frequently in ovarian cancer and are dependent upon proteasome function. Unlike 20S proteasome inhibitors (bortezomib), iRPN13 do not target immunoproteasomes as for substrate recognition they utilize PA28, which lacks RPN13, instead of 19S RP. Further Up284 promotes antigen presentation by tumor cells [26–30], a prerequisite to effective antitumor immunity.

## Materials and methods

### Cell lines and cytotoxicity assays

All cell lines were obtained from the American Type Culture Collection (ATCC) and cultured in the specified medium supplemented with 10% fetal bovine serum, 100 IU/mL penicillin, and 100 μg/mL streptomycin at 37°C in a humidified 5% CO_2_/95% air incubator. Synthesis of key compounds is described (S1 File), and >95% purity of Up284 and its analogs were confirmed by NMR and MS. To assess drug cytotoxicity, cells were seeded at 2,500 cells/well (10,000 cells/well for MM lines) in 100 μL medium in 96-well plate as triplicates and after 24 h treated with compounds for a further 72 h. To assess cytotoxicity, the cells were incubated for 2 h at 37°C according to the manufacturer’s protocol with the Thiazolyl Blue Tetrazolium Bromide (20 μL/well of 5 mg/mL stock solution in water) (Sigma, M5655) and A_570_ measured using a Benchmark Plus microplate spectrophotometer (BIO-RAD). IC_50_ was estimated using Graphpad Prism 7 software. Human and mouse liver microsomes and NADPH regenerating system solutions were purchased from Corning Life Sciences (Tewsbury, MA). Human and mouse K_2_EDTA-treated plasma was purchased from BioIVT (Westbury, NY).

### Antibodies and Western blot analyses

Cell lysate prepared in MPER (Pierce) from each sample was normalized by total protein concentration using the bicinchoninic acid assay and bovine serum albumin standards. Normalized samples were subjected to SDS-PAGE, transferred to PVDF membranes and analyzed by Western blot using antibodies specific to ubiquitin (P4D1, sc-8017, Santa Cruz), PARP (#9542, BD Pharmingen), actin (#66009, Protein Tech Group), Tubulin(#66031, Protein Tech Group), ADRM1/RPN13 (D9Z1U, #12019, Cell Signaling), USP14 (Cell Signal, # 8159), Annexin V (#559763, BD Pharmingen), and for secondary antibodies we utilized either horseradish peroxidase (HRP)-linked anti-mouse IgG or anti-rabbit IgG (GE Healthcare UK Ltd), HRP-linked streptavidin (N100, Thermo Fisher) at the recommended dilution (1:5000).

### 4UbFL reporter assays

Sub-confluent cultures of cells were transfected with 4UbFL plasmid using TransIT 2020 reagent (Mirus Bio). Cells were seeded at 10,000 cells/well in 96-well plates 48 h post transfection, and incubated with compounds or vehicle (DMSO) at the doses and for the times indicated. Luciferase activity in cell lysate was determined with a luciferase assay kit (Promega) according to the manufacturer’s instructions. Bioluminescence was measured using a Glomax Multidetection system (Promega).

### Annexin V assay

The percentage of cells initiating apoptosis was assessed with the annexin V FITC assay kit. The cells (2 × 10^5^ cells/well) were treated with the described compounds at selected concentrations for 12 h. The cells were then collected, washed with PBS, resuspended in 100 μl of annexin V and propidium iodide (PI) dual-stain solution (0.1 μg of annexin V-FITC and 1 μg of PI), and incubated 20 min in the dark. The samples were then analyzed via flow cytometry using FlowJo V10 software. All experiments were performed in triplicate and repeated three times.

### Measurement of reactive oxygen species (ROS)

Amplex Red reagent (#A12222, Thermo Scientific) combined with HRP was used to measure ROS. Briefly cells in 96 well plates (10,000 cells/well in 100 μL medium) were treated with compounds for 12 h. Next 50 μM Amplex® Red reagent plus 1 U/mL horseradish peroxidase in 50 mM sodium phosphate buffer (20 μL), pH 7.4, were added to cells and incubated for 30 minutes at room temperature. Fluorescence was measured with a fluorescence-based microplate reader (Bio Rad) using excitation at 530 ± 12.5 nm and detection at 580 ± 25 nm. Background fluorescence, determined for a no-H_2_O_2_ control reaction, was subtracted from each value. H_2_O_2_ treatment (1 h) was used as positive control.

### Biotin labeling assay

For bacterially-expressed RPN13, *ADRM1* (NM_175573)-pET28a (+) was transformed into E. coli (DE3) Rosetta 2 cells (Novagen) and cultures in LB and kanamycin at A_600_ ~0.6 were induced with 0.4 mM IPTG for 4 h at 37°C. Cell pellets were treated with BPER lysis buffer 1X cOmplete™, EDTA-free Protease Inhibitor Cocktail, and 100 μg/mL lysozyme for 30 min on ice. The lysates were clarified by centrifugation and frozen. Equal amounts of cell lysate (determined by BCA, ~40 μg) were treated with DMSO or different concentrations biotinylated compounds (Up284B, RA190B, and Up108B) for 45 min at 4°C and subjected to SDS-PAGE, transfer to PVDF membrane and probed with HRP-streptavidin. For HeLa or A2780 cells, monolayers were washed with PBS, lysed in MPER buffer (Pierce). The lysate was clarified by centrifugation and the supernatants pretreated with streptavidin-coated magnetic beads for 45 min at 4°C to deplete non-specific biotinylated proteins in the cell lysate. The beads were separated and 40 μL of the pre-cleared cell lysate was incubated with compounds (20 μM) for 45 min at 4°C, and then treated with biotinylated compounds (10 μM) for 45 min at 4°C. Next, the samples were mixed with Laemmli sample buffer (BioRad) and boiled for 5 min. The proteins were separated using a 4–15% Bio-Rad Mini-PROTEAN SDS-PAGE gel (1 h at 100 V), and transferred to PVDF membrane overnight at 4°C (24 V). The membrane was blocked with 5% (w/v) BSA in phosphate buffered saline containing 0.1% (v/v) Tween 20 (PBST) for 1 h at RT, and washed for 20 min (3X with PBST). Then the membrane was probed with HRP-streptavidin (1:10,000 in PBST) for 1 h at RT, washed for 30 min (3X with PBST), and developed using HyGLO chemiluminescent detection reagent (Denville) and visualized using a Biorad Chemidoc imager.

### Cellular Thermal Shift Assay (CETSA)

SKOV3 cells were treated with DMSO or Up284 for 30 min then washed with PBS, trypsinized, pelleted, and resuspended in PBS and aliquoted into PCR strips. Cells were incubated in a thermal cycler (Biorad IQ5 Cycler) over a gradient of temperatures for 4 min, followed by incubation at 25°C for 3 min, then snap-frozen in liquid nitrogen and subjected to three freeze–thaw cycles. Samples were briefly vortexed and centrifuged at 16,000 x *g* for 20 min at 4°C. Cleared cell lysates were resolved in SDS–PAGE followed by immunoblotting for RPN13, GAPDH, RPN2 and USP14.

### Antigen presentation

TC1 cells stably expressing Ovalbumin (TC1-OVA) were plated (125,000 cells/well) in a 6 well plate in 2 mL RPMI complete medium. After incubation for 24 h at 37°C, cells were treated with Up284, RA190 or bortezomib (0.25 μM) for 12 h. The cells were then washed with PBS, fixed and stained for 1 h at RT with APC-labeled monoclonal anti-mouse H-2K^b^ bound to SIINFEKL antibody (25-D1.16, Biolegend), and binding was analyzed by flow cytometry.

### Q-PCR to measure mRNA levels

Total RNA was isolated from cells using the RNeasy mini kit (Qiagen). Extracted RNA was normalized for concentration and reverse transcribed using an iScript cDNA synthesis kit (Bio-Rad). *CHOP10* expression levels were measured by Taqman gene expression assays with Taqman gene expression master mix (Applied Biosystems, Cat. 4369016) and run with a standard thermal cycling protocol (50°C for 2 min, 95°C for 10 min, then 40 cycles of 95°C for 15 sec and 60°C for 1 min) in a Biorad IQ5 Cycler. Calculations were done according to the Livak method and normalized to the reference gene GAPDH.

### Seahorse assay

For quantification of oxygen consumption rate (OCR), ES2 cells were seeded at 20,000 cells per well in Seahorse XF96 Analyzer plates (Agilent) in regular media and incubated at 37°C with 5% CO_2_ overnight. Cells were treated compounds for 8 h and the media was then removed, cells were washed, Seahorse medium (RPMI + 17.5 mM glucose + 1 mM pyruvate + 2 mM glutamine) was added to wells, and the plate was incubated at 37°C without CO_2_ for 1 h. The plate was then loaded into the Seahorse XF96 Analyzer for continuous readings of oxygen and pH levels were taken before and after injections of 3 μM oligomycin, 4 μM FCCP, 2 μM antimycin, and 2 μM rotenone. Oxygen consumption rate and extracellular acidification rate were analyzed using Wave 2.4 software.

### Microsomal, plasma and neat solution stability

Metabolic studies in liver microsomes (0.125 mg/mL) were conducted in 1x PBS (pH 7.4), NADPH-generating system, and 10 μM Up284 in a final volume of 250 μL. An incubation mixture with, and without NADPH-generating system, as well as a neat solution prepared without microsomes and NADPH-generating system was used. Up284 was added at the same concentration level into human and mouse EDTA plasma. All reactions were initiated with the addition of Up284. Incubations were performed in glass tubes maintained at 37°C in a shaker bath. At 0, 30, and 60 minutes, 10 μL samples were withdrawn from the reaction mixture, and 0.5 mL of acetonitrile containing internal standard was added to each. Samples were vortex-mixed and subjected to centrifugation for 5 min at 1430 *x g*. A 10 μL aliquot of the supernatant was injected onto the LC-MS/MS instrument for qualitative analysis using a temperature-controlled autosampling device operating at 5°C.

### Plasma Protein Binding (PPB)

The assay was performed in a multiple-use 96-well dialysis unit (HTD96b dialyzer). Each individual well unit consisted of 2 chambers separated by a vertically aligned dialysis membrane of predetermined pore size (MWCO 12–14 kDa). 120 μL of non-diluted plasma spiked with the compound (1 μM, final DMSO concentration 1%) was added to one chamber, and the same volume of PBS buffer pH 7.4 to the other chamber. The HTD96b dialyzer was covered with adhesive sealing film and incubated 5 h at 37°C while shaking at 100 rpm. For sample preparation, an aliquot of the contents of each chamber was mixed with the same volume of the blank opposite matrix. In order to define non-specific loss of the compound during this assay, a standard solution was created by mixing an aliquot of spiked plasma with blank buffer without dialysis. Two aliquots of the standard solution were incubated at 37°C, shaking at 100 rpm for 5 h (recovery samples). The other two aliquots were immediately diluted with acetonitrile and stored at 4°C until analysis (stability samples). All samples were diluted 5-fold with internal standard diluted in 100% methanol with subsequent plasma proteins sedimentation by centrifuging at 6000 rpm for 5 minutes. Supernatants were analyzed using LC-MS/MS. The percentage of plasma protein bound compound, recovery and stability were calculated using following equations.


Proteinbinding={(1−(arearatioinbuffer)/(arearatioinplasma)}.100%

Recovery={(arearatioinbuffer+peakareainplasma)/arearatioinrecoverysample}.100%

Stability={arearatioinrecoverysample/arearatioinstabilitysample}.100%

### Animal studies

All animal procedures were performed according to protocols approved by the Johns Hopkins University Animal Care and Use Committee, and in accordance with the AAALAC recommendations for the proper use and care of laboratory animals (protocol MO18M129, renewed as MO21M127). Four to six week old female Nude (002019), or CD1 (022) or C57BL6 (027) mice were purchased from Charles River, USA. Isoflurane anesthesia was used during imaging. The health conditions and/or criteria under which early euthanasia was implemented included, but are not limited to, general signs of distress such as hunched posture, lethargy, anorexia, dehydration, rough hair coat and/or those that are directly related to the experimental procedures e.g. loss of weight >10%, lethargy, restricted movement of limbs, distended abdomen or when the bioluminescence signal reaches 10^9^ p/s/cm^2^/sr. Palpable intraperitoneal tumor or subcutaneous tumors of 1cm diameter or greater detected by inspection and digital calipers also trigger euthanasia. Animals in distress were euthanized by carbon dioxide asphyxiation, and cervical dislocation was used to ensure death. This is an acceptable form of euthanasia for mice and in compliance with the recommendations of the Panel on Euthanasia of the American Veterinary Medical Association.

### Electroporation (EP) in vivo

A patch of CD1 mouse leg was shaved of hair and 10 μg 4Ub-FL plasmid in 20 μL of PBS was injected into the *quadriceps femoralis* muscle followed immediately by injection of the 2 Needle Array to 5 mm depth encompassing the injection site and square wave EP (ElectroSquarePorator 833, BTX-2 Needle array 5mm gap, Harvard apparatus) delivered as eight pulses at 106V for 20 ms with 200 ms intervals. One day post-EP, mice were anesthetized with isoflurane, injected IP with luciferin (0.3 mg in 100 μL water) and optical imaging was performed to determine basal level luciferase expression. Images were acquired for 10 min with a Xenogen IVIS 200 (Caliper, Hopkinton, MA). Equally sized areas were analyzed using Living Image 2.20 software.

### Repeat dosing toxicity study

The mice were divided into three groups consisting of three female CD1 mice each. The mice of Groups 1, 2, and 3 were repeatedly IV dosed with Vehicle (25% 2-β-HPCD in water), 20 mg/kg of Up284 in Vehicle, or 1 mg/kg of bortezomib in saline, respectively, with 72h intervals (on the days 0, 3, and 6 of the 7 day study). Animals were observed for mortality and signs of gross toxicity at 30 min, 1 h, 2 h, 4 h, and 6 h after compound administration, and thereafter daily. Terminal blood sampling for hematological and serum biochemical analysis was performed on Day 7 of the study, 24 h after the last dosing. Up284 was dissolved in 25% 2-HPβCD in water at a concentration of 4 mg/mL for the dose of 20 mg/kg. Bortezomib was dissolved in physiological saline at 0.2 mg/mL for the dose of 1 mg/kg. Terminal bleeding and euthanasia were performed on Day 7, 24 h after the third dosing. Blood collection was carried out after 6 h fasting. Animals were injected IP with 2,2,2-tribromoethanol (200 mg/kg) and the blood was collected from orbital sinuses into dry microtainers without anticoagulant. For the serum collection, the tubes with collected blood were held at RT for 15–60 min after blood drawing to form a clot, and then were centrifuged at 9000 rpm for 20 min at 4°C. The collected serum was frozen within 60–80 min after blood drawing and was stored at -20°C for further clinical chemistry analysis. For the hematological study, 25 μL of fresh blood was quickly mixed with an equal volume of 0.36% solution of K_2_EDTA in saline. Assessment of the hematological parameters was performed using the BIO TE hematology analyzer MCL-3124 and consumable reagents (Cormay) within 2 h of the blood draw. For clinical chemistry analyses, ASAT, ALAT, ALP, LDH, GGT, CK, Creatinine, Urea and TP were determined in the serum using commercial kits according to the manufacturer’s instructions.

### ES2-luc xenograft model

Female Nude mice were inoculated IP with 1x10^6^ ES2-luc in 100 μL PBS. At day 3, mice were imaged for basal level luminescence activity with a Xenogen IVIS 200 after IP injection with luciferin (0.3 mg in 100 μL water). Mice were randomized into groups and treated IP with compounds or vehicle (25% (*w*/*v*) β-Hydroxypropylcyclodextrin in water), and imaged again on days indicated.

### ID8-Defb29/VegfA-luc syngeneic model

Female C57BL6 mice were inoculated IP with 3x10^5^ ID8-Defb29/VegfA-luc cells [31] in 100 μL PBS. At day 3, mice were imaged for basal level luminescence activity with a Xenogen IVIS 200 after IP injection with luciferin (0.3 mg in 100 μL water). Mice were randomized into groups and treated IP with compounds or vehicle (25% (*w*/*v*) β-Hydroxypropylcyclodextrin in water), and imaged again on days indicated.

### Spontaneous genetically-engineered mouse model (GEMM) of ovarian cancer

To induce IP tumor formation in immunocompetent mice, oncogenes (shp53, AKT, c-Myc), luciferase and the Sleeping Beauty (SB) transposase (10 μg/plasmid) were diluted in 500 μL of PBS and injected IP into female C57BL/6 mice [32]. The mice were anesthetized by intramuscular injection of ketamine. The plasmid-injected mice were electroporated (EP) by the BTX ECM 830 square wave EP generator (BTX) (5 pulses, 200 V for 100 ms/pulse, 100 ms intervals between each pulse), the caliper electrode (BTX) was held on the waist of mouse. The mice were followed by IVIS imaging weekly for tracking tumor growth. The mice were sacrificed when the bioluminescence signal reached 10^9^ photons/sec/cm^2^/sr, or had enlarged abdomens due to the production of ascites, or had health conditions and/or criteria under which early euthanasia was required by the protocol (see above). To track peritoneal tumor growth, bioluminescence imaging was performed by the IVIS spectrum2000 (PerkinElmer) as the plasmid expressing SB transposase also produces luciferase. Briefly, the mice were injected IP with D-luciferin (GoldBio). After 10 min, the mice were anesthetized with isoflurane, and imaged by the IVIS Spectrum under the auto exposure mode. Bioluminescence in the peritoneal region was quantified as total photon counts/sec/cm^2^/sr using Living Image 3.0 Software (Perkin Elmer).

### Statistical analyses

Results are reported as mean ± standard deviation (s.d.). Statistical significance of differences was assessed by two-tailed Student’s *t* using Prism (V.5 Graphpad, San Diego, CA) with the level of significance set at p≤0.05. Survival was summarized using Kaplan–Meier methods and compared using log-rank tests. Combination indices (CI) were calculated using Synergy finder.

### Pharmacokinetics

For single dose pharmacokinetic studies, plasma samples (40 μL) were mixed with 200 μL of a water-methanol mixture 1:9, v/v containing the internal standard elacridar at 400 ng/mL. After mixing by pipetting and centrifuging for 4 min at 6000 rpm, 1 μL of each supernatant was injected into LC-MS/MS system. Chromatographic separation was achieved with a Luna C18(2) (50 x 2 mm, 5μm; Phenomenex) column at 30°C using gradient elution over a 2.2 min analytical run time. Mobile phase A was acetonitrile/water (5:95, v/v) containing 1% formic acid, and mobile phase B was acetonitrile containing 0.1% formic acid. The gradient was initiated with mobile phase B at 15% and was held at 15% for 0.9 minutes with a flow rate of 0.4 mL/min, then increased to 100% and held for 0.1 minutes, and finally returned back to 15% mobile phase B and allowed to equilibrate until 2.2 minutes. An AB Sciex 3000 triple quadrupole mass spectrometer operated in positive electrospray ionization mode was used for the detection of Up284. The lower limit of quantitation (LLOQ) in plasma was 50 ng/mL.

For the stability studies and biodistribution studies, plasma or tissue homogenate was extraction using acetonitrile containing the internal standard RA413S (a structural analogue) at 10 ng/mL. Tissue homogenates were prepared at a concentration of 200 mg/mL in PBS. After vortex-mixing and centrifuging for 5 min at 2700 rpm. Chromatographic separation was achieved with a Zorbax XDB C18 (50 x 2.1 mm, 3.5μm; Agilent) column at RT using gradient elution over a 4.1 min analytical run time. Mobile phase A was water containing 0.1% formic acid, and mobile phase B was acetonitrile containing 0.1% formic acid. The gradient started with mobile phase B at 20% and was held at 20% for 0.5 min with a flow rate of 0.3 mL/min, then increased to 100% over 0.5 minutes and held for 2.0 min, and finally returned back to 20% mobile phase B and allowed to equilibrate until 4.1min. An AB Sciex 5500 triple quadrupole mass spectrometer operated in positive electrospray ionization mode was used for the detection of Up284. The lower limit of quantitation (LLOQ) in plasma was 10 ng/mL.

### Pharmacokinetic method analysis

Pharmacokinetic parameters were calculated from mean concentration-time data using non-compartmental methods in Phoenix WinNonlin version 8.3 (Certara, Princeton, NJ). Concentrations after the time 0 sample that were below the LLOQ were imputed with ½ that value. Any concentration that was determined to be an outlier by the Grubbs’s outlier test were excluded from analysis. The maximum plasma concentration (C_max_) and time to C_max_ (T_max_) were the observed values. The AUC_last_ was calculated using the log-linear trapezoidal method. AUC was extrapolated to infinity (AUC_INF_) by dividing the last quantifiable concentration by the terminal disposition rate constant (λ_z_). The λ_z_ was determined from at least 3 points on the slope of the terminal phase of the concentration-time profile. The terminal half-life (T_1/2_) was determined by dividing 0.693 by λ_z_. Apparent clearance (Cl/F) was calculated by dividing the dose administered by AUCINF. Apparent volume of distribution (V/F) was calculated by dividing Cl/F by λ_z_. If the percent AUC extrapolated was >20% or the r^2^ of λ_z_ was <0.9, the AUC_INF_, Cl/F, T_1/2_ and V/F were not reported. The oral and IP bioavailability was calculated as:

F(%)={DoseIV×AUC(0−∞)PO/DosePO×AUC(0−∞)IV}.100%

The Method of Bailer was used to estimate the variance of AUC given the calculated variance of the mean concentration at each time point [33]. A pairwise comparison utilizing Z test was used to determine whether there was a significant difference between Up284 exposures as expressed by AUC _last_ [34]. In all cases, p < 0.05 was considered statistically significant.

## Results

### Development of Up284

**Fig 1:**
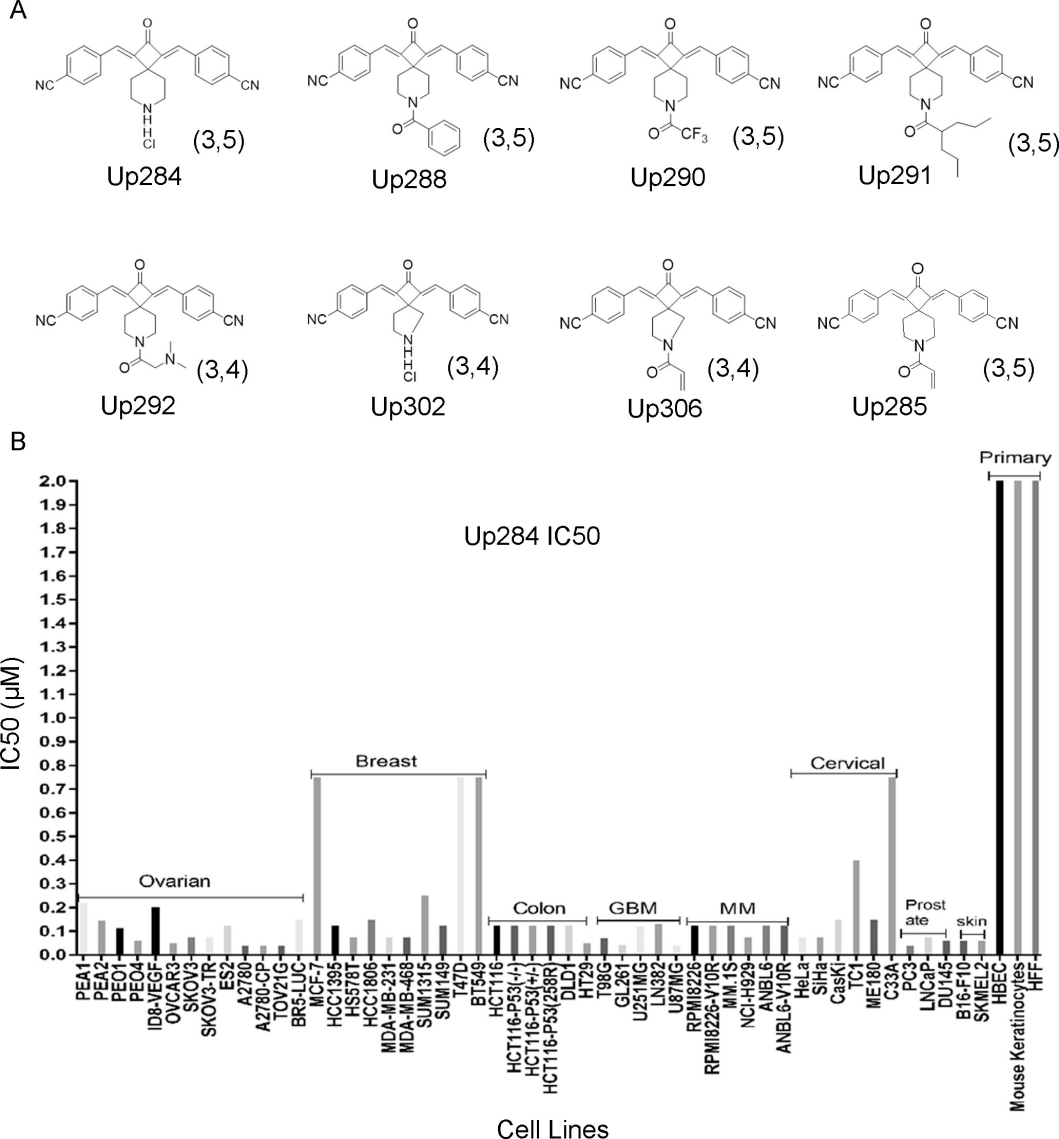
Cytotoxicity of Up284 against cell lines of diverse cancer origin. A. Chemical structures of key compounds. B. The cytotoxicity of Up284 for a broad panel of cancer cell lines. Briefly each cell line (~2500–10,000 cells/well depending on the cell type) was treated with titrations of Up284 in triplicate and incubated for 72 h, and the cell viability was then measured using MTT reagent. The data were plotted and IC_50_ analyzed by curve fitting in Graphpad Prism.

Analogs of Up109 were rationally designed to improve solubility and other key drug properties with a focus on the electron withdrawing groups (EWG), and the central ring nitrogen substituents. In designing more drug-like iRPN13, we screened out compounds with PAINS features, using both the ‘bad apple’ algorithm [5] and the SwissADME web tool [6]. The functional EWG (-NO_2_) and acryloyl moieties in Up109 were considered likely to contribute toxicities and poor solubility, and thus nitrile (-CN), another strong EWG, was substituted on both sides of the rings (Fig 1).

Different substituents were examined on the central ring nitrogen atom, and these analogs were screened for cancer cell cytotoxicity (**Table 1**). While inclusion of acryloyl provided the most potent compound of the series, Up285, its removal did not profoundly reduce the cytotoxicity, suggesting the potential of Up284. Interestingly, the 3,5 spiro ring analogs were more potent than the 3,4 equivalents. Other central ring nitrogen substituents tested only compromised drug cytotoxicity (**Table 1**). Furthermore, Up284 also possesses a strong score (5) for drug-like characteristics based on the Lipinski, Ghose, Veber, Egan and Muegge rules, along with good synthetic accessibility. Hence Up284 was advanced for further study.

**Table 1:** Structural features of substituted cyclic piperidones and their cytotoxicity. Structures and synthesis of the compounds is described in S1 File. Toxicity to HeLa or SKOV3 cells by IC50 by MTT assay is shown in μM. * as amide.

Compound	Aromatic substituents	R3	[Ring Size]	HeLa	SKOV3
Up104	4NO_2_	H	3,5	0.18	0.35
Up108	3,4-diCl	H	3,5	>1.25	>1.25
Up109	4NO_2_	Acryloyl	3,5	0.045	0.052
Up111	3,4-diCl	Acryloyl	3,3	>1.25	>1.25
**Up284**	**4CN**	**H**	**3,5**	**0.04**	**0.071**
**Up285**	**4CN**	**Acryloyl**	**3,5**	**0.025**	**0.06**
Up288	4CN	benzoyl	3,5	0.28	0.39
Up290	4CN	CO-CF3	3,5	0.15	0.29
Up291	4CN	Valproic	3,5	0.25	0.42
Up292	4CN	N,N-Dimethylglycine*	3,5	0.23	0.35
Up302	4CN	H	3,4	0.08	0.17
Up306	4CN	Acryloyl	3,4	0.05	0.12
Up310	4CN	CH2-C≡C—	3,5	0.5	0.75
Up323	3F, 4CN	H	3,5	0.05	0.09
Up284-NMe	4CN	Methyl	3,5	>2.5	>2.5

### Up284 inhibits proliferation of cancer cells of diverse origin

To determine whether Up284 cytotoxicity is specific for particular cancer types, a panel of cell lines derived from various cancer types and normal tissues were treated with Up284 for 72 h and IC50 determined. Up284 demonstrated potency against the growth of most of the cancer types, suggesting broad applicability, but relatively spared normal cells, implying a significant therapeutic window (Fig 1B). However, several cell lines were less sensitive to Up284, notably MCF7, T47D, BT549 and C33A. This panel included cell lines isolated from patients with drug-resistant disease (PEA2, PEO4) or pre-treatment (PEA1, PEO1) [35] that showed similar sensitivity to Up284. Likewise, parental RPMI8226 and ANBL6 multiple myeloma cell lines and those selected *in vitro* for resistance to bortezomib (-V10R) were similarly very sensitive to Up284. HCT116 cells, regardless of *Tp53*^-/-^, *Tp53*^+/-^ or *Tp53*^285R^, were similarly sensitive suggesting Up284-induced cell death occurs independently of Tp53 signaling (Fig 1B).

### Up284 covalently binds RPN13

**Fig 2:**
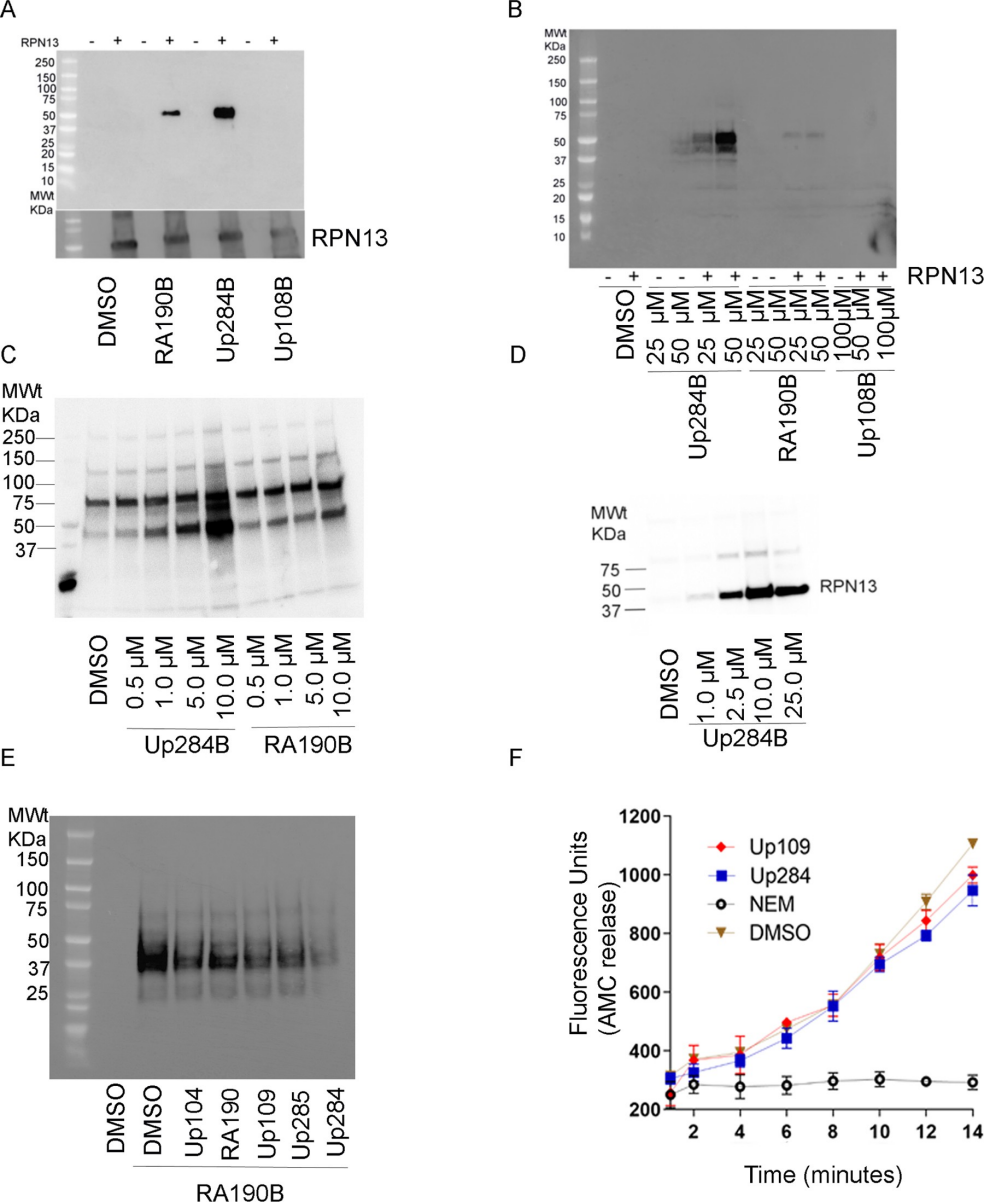
Biotin labeling of Up284 and its binding to RPN13 in cell lysates. A) *E*. *coli* Rosetta 2(DE3) bacterial cell lysate expressing RPN13 (tagged with 6His at both termini, expected MW 46.8KDa) was treated with biotinylated RA190 (RA190B), Up284 (Up284B) or inactive compound Up108 (Up108B) at 50 μM for the period of 45 min at 4°C and subjected to SDS-PAGE and transfer to PVDF membrane. The membrane was probed with HRP-Streptavidin (upper panel) and anti-RPN13 (lower panel). B) Same as in A, but examining dose dependency of labeling of RPN13 in 2DE3 bacterial cell lysate expressing 6His-RPN13. C) A2780 cell lysate was pretreated with streptavidin beads for 45 min at 0°C to remove non-specific biotinylated proteins. Next, the lysates were treated with Up284B or RA190B at 0.5 μM to 10 μM for 45 min at 0°C and subjected to SDS-PAGE, PVDF membrane transfer analysis and probed with HRP-streptavidin. D) As in C, but lysate of human ovarian cancer cell line ES2 was treated with Up284B (1–25 μM) for 45 min at 0°C. E) As in C, but lysate of HeLa cells was pre-cleared with streptavidin beads. Aliquots of pre-cleared lysate were pre-treated with the indicated compounds (20 μM) for 45 min at 0°C and then each incubated with RA190B (40 μM) for 45 min at 0°C. To detect labeling, the lysates (40 μg) were subject to SDS-PAGE, PVDF membrane transfer, and then probed with HRP-streptavidin. The membrane was stained with Ponceau solution to confirm equal loading. F) SKOV3 cells were treated with the indicated compounds (5 μM) for 1 h, and then total cellular DUB activity determined by measuring Ub-AMC cleavage over time. NEM (1 mM) used as positive control.

Earlier we demonstrated that biotinylated RA190 (RA190B) covalently binds to Cys88 of RPN13 [19]. Taking a similar approach, the central ring nitrogen of Up284, and that of an inactive spiro analog Up108, were each modified with biotin (Up284B and Up108B respectively) and their capability to bind covalently to RPN13 examined. Briefly, *E*. *coli* DE3 were transformed with an expression vector for 6His tagged RPN13 (MW 46.8KDa) and its expFADRM1ression induced. A crude detergent lysate was incubated with 50 μM of RA190B, Up284B or Up108B for 45 min at 4°C. Next, the samples were separated by SDS-PAGE, transferred to a PVDF membrane, probed with HRP-linked streptavidin and binding visualized by chemiluminescent substrate. *E*. *coli* DE3 bacterial lysate was used as a negative control. Up284B demonstrated robust labeling of 6His-RPN13, whereas the inactive molecule Up108B (with dichloro moieties on the aromatic rings) failed to react detectably (Fig 2A, Upper panel). The membrane was stripped and probed with RPN13 antibody to confirm its expression (Fig 2A, lower panel). Labeling was dose dependent, and Up284B labeled 6His-RPN13 more strongly than RA190B (Fig 2B).

Next, the covalent binding profile of Up284B in detergent lysates of the human cancer cell lines A2780 (Fig 2C) and ES2 (Fig 2D) was assessed. Up284B bound with specificity to a cellular protein of 42kDa, a molecular weight consistent with that of RPN13 (42.1KDa), and more strongly than seen for RA190B.

### Up284 competes RA190B binding to 42KDa cellular target, RPN1

3A competition assay was performed by pre-treating HeLa cell lysate with non-biotinylated compounds (5 μM) for 45 min at 4°C, including RA190 as a ‘cold’ competitor and both bortezomib and DMSO vehicle as negative controls. These lysates were then probed with RA190B (20 μM) for 45 min at 4°C, separated by SDS-PAGE, transferred to a PVDF membrane and probed with HRP-linked streptavidin. Upon chemiluminescent visualization, it was evident that all the cytotoxic spirocyclic compounds Up104, Up109, Up284 and Up285 competed like RA190 for RA190B binding to the 42KDa cellular protein (Fig 2E). In contrast the non-cytotoxic Up111 (Table 1) did not compete, as seen with the negative controls bortezomib and DMSO (Fig 2E).

Cellular deubiquitinating enzymes (DUBs) are proteases that remove ubiquitin, or ubiquitin-like molecules, or remodel ubiquitin-chains on target proteins, and many contain active site cysteines [36]. To examine whether Up284 non-specifically inhibits cellular DUB enzymes, total cellular DUB activity was measured using the substrate Ub-7-amino-4-methylcoumarin (Ub-AMC) (Fig 2F) in SKOV3 cells treated 1 h with 5 μM Up109 or Up284 and lysed. No reduction in total lysate DUB enzymatic activity was noted with either compound; N-ethylmaleimide (NEM, 1 mM) served as a positive control for DUB inhibition.

### Up284 stabilizes RPN13 in Cellular Thermal Shift Assay

**Fig 3:**
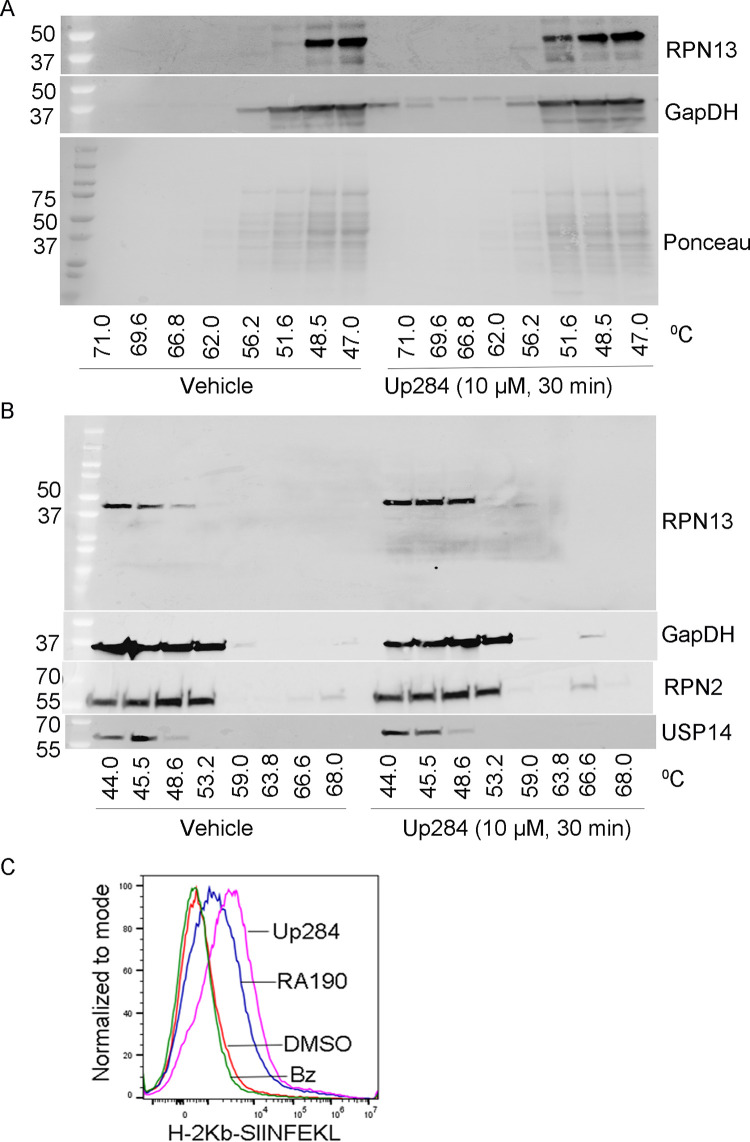
Up284 thermostabilizes RPN13 and promotes antigen presentation. A and B) SKOV3 cells were treated with DMSO or 10 μM Up284 for 30 min then washed with PBS, trypsinized, pelleted, and resuspended in PBS supplemented with Halt protease inhibitor cocktail (Thermo Scientific). Resuspended cells were aliquoted into PCR strips. Cells were incubated in a thermal cycler (Bio-Rad) over the indicated gradients of temperature for 4 min, followed by incubation at 25°C for 3 min. Cells were snap-frozen in liquid nitrogen and subjected to three freeze–thaw cycles. Samples were briefly vortexed and centrifuged at 16,000 *g* for 20 min at 4°C. Cleared cell lysates were mixed with a 1/3 volume of 6×Laemmli sample buffer. After boiling, cell lysates were resolved in SDS–PAGE followed by immunoblotting for RPN13, GAPDH, RPN2 and USP14. C) TC1-OVA cells were plated (125,000 cells/well) in a 6 well plate in 2 mL/well in complete medium. After incubation for 24 h at 37°C, cells were treated with Up284, RA190 or bortezomib (0.25 μM) or vehicle for 12 h. The cells were then washed with PBS, fixed and stained for 1 h at RT with APC-labeled monoclonal anti-mouse H-2K^b^ bound to SIINFEKL antibody (25-D1.16, Biolegend), and binding was analyzed by flow cytometry.

Cellular Thermal Shift Assay (CETSA) assesses thermal stabilization of proteins upon ligand binding and is used extensively to detect drug interactions by implementing a thermal shift in the cellular context [37, 38]. The assay involves drug treatment of cells, heating to denature and precipitate proteins in a thermocycler with a specified gradient of temperatures, cell lysis by freeze/thaw cycles, and the separation of cell debris and aggregates from the soluble protein fraction by centrifugation. Whereas unbound proteins denature and precipitate at elevated temperatures, ligand-bound proteins remain in solution. The proportion of individual proteins remaining in solution can be detected by quantitative Western blotting. In repeated experiments, treatment of SKOV3, 10 μM Up284 for 30 min stabilized RPN13 to heat treatment compared to vehicle (DMSO) treated samples, such that it resisted denaturation to higher temperatures (Fig 3A and 3B). There was no impact on other control proteins tested, including a house keeping protein (GAPDH) and proteasome subunits (RPN2 and USP14). This data is consistent with on-target Up284 activity against RPN13 and supports its specificity.

### Up284 enhances antigen presentation in vitro

The approved 20S proteasome inhibitors block the function of both the constitutive proteasome and the immunoproteasome [39]. This likely accounts for their side effects of thrombocytopenia and neutropenia because the immunoproteasome is constitutively expressed in hematopoietic cells [40]. Unlike the constitutive proteasome, the immunoproteasome does not require RPN13 (the 19S RP is replaced by PA28 that does not contain RPN13), and this may account for the absence of the above side effects in mice treated with iRPN13 [40, 41]. Notably, the immunoproteasome is central in antigen presentation, and thus the impact of Up284 versus bortezomib upon presentation of an endogenous model antigen, ovalbumin (OVA), was examined in a murine cervical cancer model cell line, TC1, stably expressing OVA. TC1-OVA cells were treated with Up284, RA190 or bortezomib (0.25 μM) for 12 h. The cells were then fixed and stained with a monoclonal antibody specific for the OVA MHC class I epitope (SIINFEKL) when presented on H-2Kb, and binding was analyzed by flow cytometry (Fig 3C). Indeed, Up284 enhanced MHC class I antigen presentation by the TC1-OVA cells, whereas bortezomib did not (Fig 3C). This finding suggests inhibition of the constitutional proteasome by RA190 and Up284 causes an accumulation of ovalbumin, but neither inhibits the immunoproteasome responsible for processing the ovalbumin for MHC class I presentation. By contrast, bortezomib inhibits both the constitutional proteasome and immunoproteasome such that ovalbumin accumulates but the excess is not processed for MHC class I presentation.

### Up284 cytotoxicity is associated with ER stress and mitochondrial dysfunction

**Fig 4:**
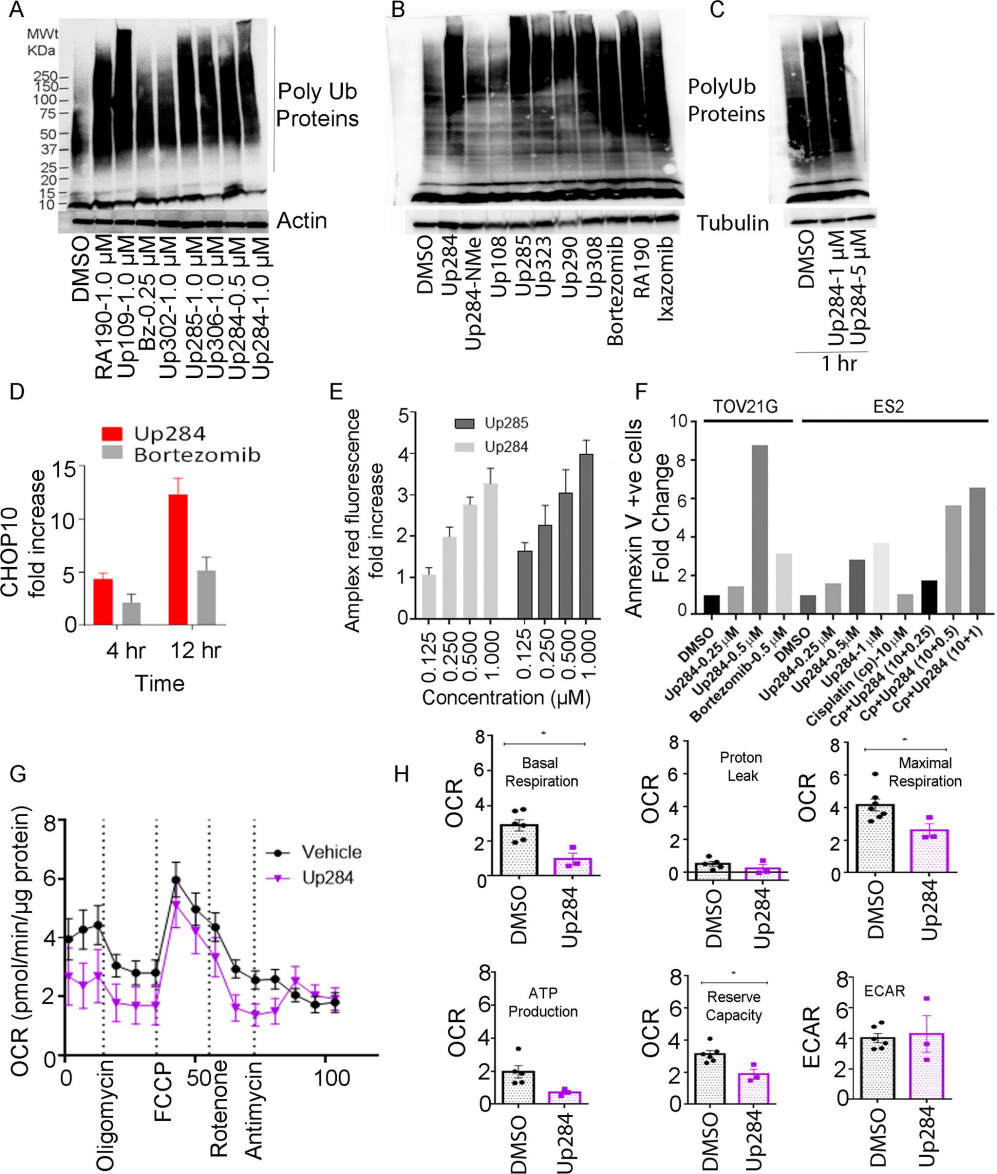
Downstream effects of Up284 treatment. A) ES2 cells were treated with each compound for 12 h at indicated doses and then lysates subjected to Western blot analysis and using anti-ubiquitin antibody, or anti-actin as the loading control. B) As in A, but ES2 cells were treated for 4 h with 1 μM of the indicated compounds. Lysates were analyzed by Western blot using anti-ubiquitin antibody, or anti-tubulin as the loading control. C) As in B, but HeLa cells were treated for 1 h with 1 μM or 5 μM Up284. D) ES2 cells were treated with DMSO, or 1 μM Up284 or Bortezomib for 4 h. Total RNA was isolated and analyzed for *CHOP10* expression by qRT-PCR. E) ES2 cells were treated with Up284 or Up285 at the indicated doses for 12 h and cellular levels of ROS were measured using Amplex Red/HRP fluorescence (571/585 nm) measurement. F) Ovarian cancer cell lines TOV21G or ES2 treated with compounds (Up284, Bortezomib, cisplatin or combination) at indicated doses for 12 h. The cells were stained with PE-Annexin and 7-AAD for 20 min in the dark and analyzed by flow cytometry. G-H) Impact on mitochondrial was assessed using the Seahorse XF instrument in ES2 cells upon treatment with Up284 (0.5 μM) or DMSO for 12 h. G) Oxygen consumption rate (OCR) was measured following sequential additions of Oligomycin, FCCP, Rotenone and Antimycin A in to the culture media. H) OCR and extracellular acidification rate (ECAR) were measured by Seahorse XF analyzer after treatment with Up284 (0.5 μM) or DMSO. Data are representative of 3 independent experiments, and 9 biologic replicates per conditions used.

Like RA190 and Up109, Up284 and its cytotoxic analogs (Table 1) also caused profound accumulation by 12 h of HMW polyUb proteins in ES2 cells (Fig 4A), and as early as 1 h (Fig 4B and 4C), to a molecular weight greater than seen with bortezomib. Depletion of mono- and di-ubiquitin levels was evident at 12 h, but not after only 4 h of treatment (Fig 4A and 4B). This implies Up284, unlike bortezomib, blocks a DUB activity. Up284 does not globally inhibit cellular DUBs (Fig 2F). This suggests Up284 may instead indirectly inhibit a proteasome-associated DUB by blocking substrate recognition, such as UCH37 whose activity is dramatically enhanced upon RPN13 binding to polyubiquitinated protein substrate [42].

Accumulation of these HMW polyUb proteins by Up284 promotes UPR signaling, including rapid upregulation of CHOP10 mRNA levels in OVCAR3 cells (Fig 4D). Furthermore, induction of reactive oxygen species (ROS) was detected using Amplex red fluorescence after treatment of ES2 cells with compounds (Fig 4E), a mechanism that contributes to bortezomib-induced cytotoxicity [43, 44]. Early apoptosis was evident in two ovarian cancer cells (TOV21G and ES2) by assessing Annexin-V labeling on the cell surface; Up284 increased the fraction of Annexin-V positive cells by 12 h of treatment (Fig 4F), consistent with rapid onset of early apoptosis.

Since ubiquitin signaling plays a critical role in DNA damage repair, and Up284 causes a depletion in the monoubiquitin pool (Fig 4A), Up284 was tested for potential synergy with cisplatin. In comparing apoptosis induced at 12 h by Up284 alone or in combination with cisplatin, there was no indication of synergy at this early time point, and indeed cisplatin alone had minimal impact (Fig 4F). By contrast, when examining checkerboard combinations of Up284 and cisplatin for cytotoxicity at 72 h, there was clear evidence of synergy in several cell lines including PEA2 and PACS (S1 Fig). We also observed synergy with Up284 and doxorubicin combination in SKOV3 cells (S2 Fig).

The rapid onset of apoptosis caused by proteasome inhibitors has also been associated with compromised mitochondrial function [45]. To examine its impact on cellular energetics, we examined in ES2 cells whether changes in oxidative phosphorylation (OXPHOS) or glycolysis occurred with Up284 treatment (Fig 4G and 4H). Mitochondrial electron transport chain (ETC) dysfunction, oxygen consumption rate (OCR) and anaerobic acid production via extracellular acidification rate (ECAR) were assessed using a Seahorse XF flux analyzer. OCR was measured at baseline and after addition of oligomycin, a complex V inhibitor, followed by an uncoupler, carbonilcyanide p‐triflouromethoxyphenylhydrazone (FCCP), then rotenone (complex I inhibitor), and finally antimycin (complex III inhibitor) (Fig 4G). Upon 12 h of Up284 treatment there was a significant decrease in OCR, both in basal and respiration stimulated with FCCP, and ATP production. Up284 did not impact proton leakage. Overall OXPHOS inhibition by Up284 significantly reduced the mitochondrial energy reserve in ES2 cells, which is essential for cell survival. Furthermore, Up284 treatment showed a decrease in mitochondrial ATP production by inhibiting the OXPHOS. A trend for reduction in ECAR, a surrogate measure of glycolysis, was observed after 24 h Up284 treatment (Fig 4H). These observations suggest that Up284 affects ES2 cancer cells by inhibition of glycolysis and OXPHOS, thereby ultimately reducing mitochondrial ATP production as previously shown with RA413S, a bis-benylidine piperidone iRPN13 candidate [46].

#### Stability of Up284 in plasma and liver microsomes

As a prelude to in vivo studies, the he stability of Up284 in murine and human plasma was first assessed (S1 Table). Up284 was stable in murine and human plasma (<10% degradation) after incubating for 1 hour and analysis by HPLC-MS system. To determine binding capabilities of Up284 to mouse plasma proteins, LC-MS/MS analysis was performed by spiking 1 μM Up284 into plasma and dialyzing 5h against buffer to seek equilibrium. Up284 showed 98% mouse plasma protein binding (S2 Table). Up284 stability in murine and human liver microsomes, either with and without nicotinamide adenine dinucleotide phosphate (NADPH), was also examined (S3 Table). Up284 was degraded in the absence and presence of NADPH. There was ~50% degradation in human or mouse liver microsomes without NAPDH with an additional ~10% degradation in the presence of NAPDH suggesting minimal NADPH-dependent metabolism (S3 Table). These findings are similar to those for RA190, but RA183 was more rapidly broken down in human and mouse microsomes [47].

### Up284 tolerability in mice

Pilot studies assessed the safety and tolerability of Up284 only treatment in female 9 week old CD1 mice (n = 3/dose) by delivering single, ascending doses of Up284 dissolved in 25% aqueous β-hydroxypropylcyclodextrin (S4 Table) delivered as 5 mL/kg intravenously (IV), 10 mL/kg intraperitoneally (IP), or 10 mL/kg per orally (PO) with weight measurements daily (S5 Table). Mice were dosed on day 1 and clinical condition recorded at 0.5 h, 2 h, 4 h, 6 h, and days 1–7 (S6 Table). Mice reaching day 7 were bled for hematologic and clinical chemistry analyses (S7 Table). The MTD for a single IP of dose Up284 was 40 mg/kg. No MTD was reached even at the maximum feasible doses (MFD; due to solubility and injection volume limitations) of 60 mg/kg for IV delivery and 200 mg/kg by PO delivery; at these doses there was no significant change in mean body weight over 1 week post dosing, or in average hematologic or serum chemistry parameters at day 7 as compared to vehicle-treated mice.

In a repeat dose study, groups (n = 3/condition) of female CD1 mice were treated with 3 cycles of bortezomib (1 mg/kg IV every 3 days, a typical therapeutic dose), Up284 (20 mg/kg, IV every three days) or vehicle (25% β-hydroxypropylcyclodextrin, IV) (S8 Table). Body weight determinations (S9 Table) and clinical observations were made over 7 days (S10 Table) and showed no significant differences to vehicle-treated controls. Blood samples collected 24 h after the third dose were subject to hematologic (S11 Table) and chemistry panel analysis (S12 Table). Up284 did not significantly change any blood parameters compared to vehicle (S11 Table) with the exception of slight reductions in red blood cell numbers, hemoglobin and hematocrit that remained within normal range. Bortezomib treatment likewise significantly reduced red blood cell numbers, hemoglobin and hematocrit compared to vehicle-treated mice albeit within normal ranges. However, in addition, bortezomib significantly reduced granulocyte and platelet counts, consistent with the clinical experience. The clinical chemistry parameters of the Up284, or bortezomib, -treated mice were not significantly different to vehicle control, with the exception of a reduction in alkaline phosphatase levels (S12 Table).

Dosing of Up284 was tested in female Balb/c and C57BL/6 mice (n = 3/group) every third day and after 3 doses the MTD for Up284 was 40 mg/kg for IP delivery (60 mg/kg was toxic) suggesting that its MTD is independent of mouse strain.

### Assessment of Up284 in vivo on-target activity using proteasome reporter gene

**Fig 5:**
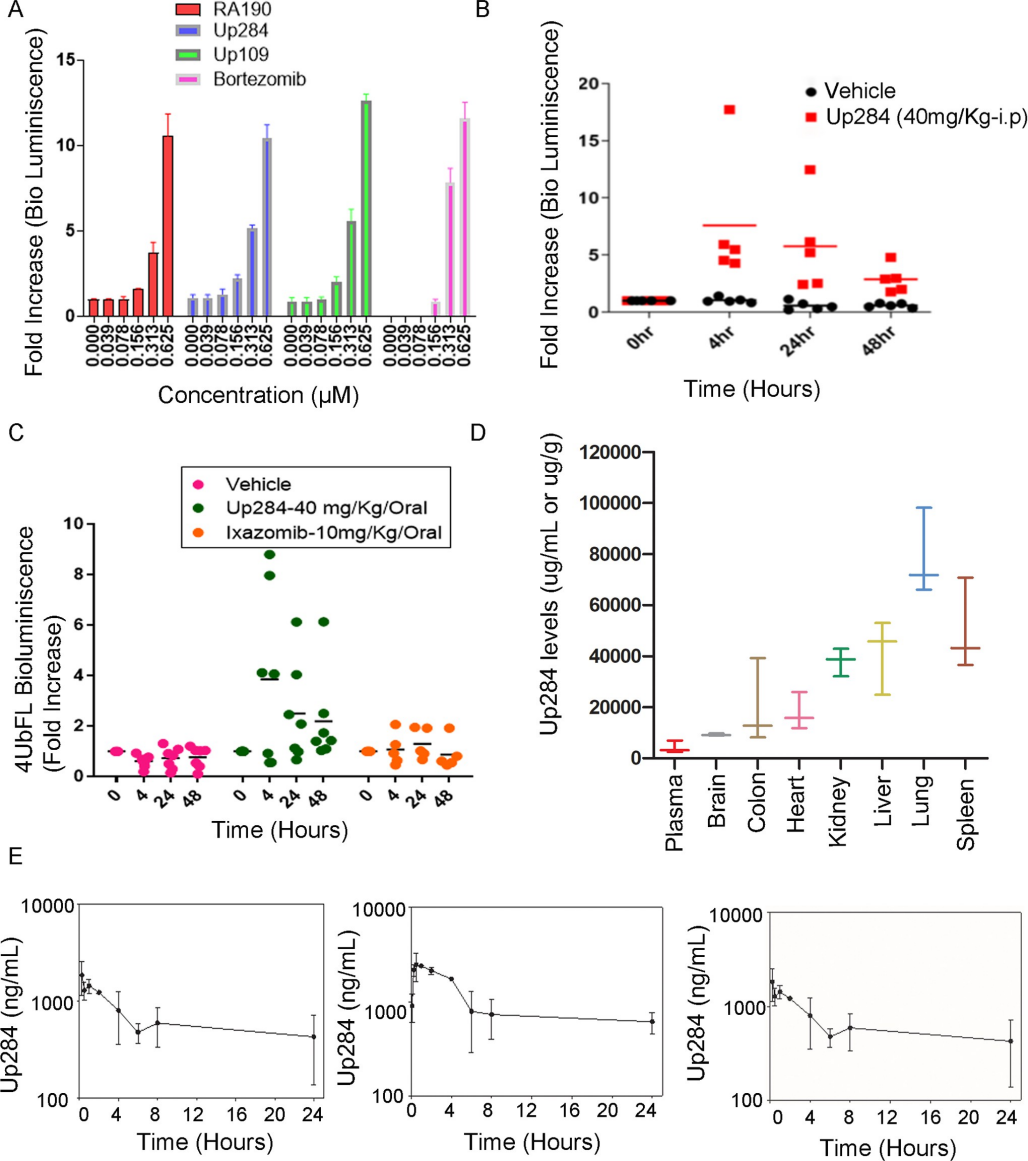
Pharmacodynamics and Pharmacokinetics of Up284 in mice. A) ES2 cells stably transduced with the 4UbFL reporter plasmid were treated with compounds at indicated doses for 4 h. The cells were then washed, lysed and their luminescence measured by a luminometer after addition of luciferin. B) Female CD1 mice electroporated with 4UbFL plasmid in their leg muscle. After 48 h, the mice were injected with luciferin, imaged by IVIS for their basal bioluminescence and randomized into two groups of five. The groups of mice were treated IP with one dose of either vehicle or Up284 (40mg/kg) and imaged for bioluminescence at 4 h, 24 h and 48 h by IVIS. C) As in B, CD1 mice (n = 5) were treated PO with one dose of vehicle, Up284 (40mg/kg), or Ixazomib (10mg/kg). D) Up284 tissue distribution. Female CD1 mice (n = 3) were treated with one IV dose of Up284 (40mg/kg) and euthanized after 24 h with collection of plasma and tissues including brain, colon, heart, kidney, liver, lung and spleen. Up284 levels in tissue samples were analyzed by HPLC. E) Up284 pharmacokinetics. Male CD1 mice (n = 4) were treated with Up284 IV (5mg/kg), IP (20mg/kg) or PO (50mg/kg). Blood was collected at indicated time points thereafter and analyzed for Up284 levels by HPLC.

As seen with RA190 and bortezomib, *in vitro* treatment of ES2 cells stably expressing the 4UbFL reporter for 4 h with Up284 resulted in its dose-dependent stabilization as indicated by a fold increase in luminescence over baseline (Fig 5A). To test for proteasome inhibition by Up284 *in vivo*, leg muscle of live CD1 mice was transduced with the 4UbFL reporter DNA construct by electroporation. Two days later, luciferin was injected IP and the baseline enzymic activity of luciferase in the transduced muscle tissue was visualized as bioluminescence within this region of interest (ROI) using an IVIS imager. A control group (n = 5) of mice was treated IP with vehicle alone and another group (n = 5) treated IP with a single dose of Up284 (40 mg/kg). After 4h, 24h and 48h the mice were again injected with luciferin, imaged and bioluminescence was quantified (Fig 5B). Up284 significantly enhanced bioluminescence levels over >48h. In another experiment, Up284 was delivered PO once at 40mg/kg to CD1 mice (n = 7) and compared with licensed orally-available 20S proteasome inhibitor ixazomib at 10 mg/kg (7.5mg/kg is therapeutic dose [48]). Surprisingly, Up284 better stabilized the reporter than ixazomib, suggesting better accessibility to solid tissue like muscle (Fig 5C).

### Pharmacokinetics and tissue distribution of Up284 in mice

For a pilot assessment of its tissue distribution, CD1 mice were injected with one dose of Up284 (40 mg/kg, IV) and tissues collected 24 h later. Up284 levels were analyzed in all major tissues (brain, colon, heart, kidney, liver, lung and spleen) by liquid chromatography–mass spectrometry (LC–MS/MS). Up284 accumulated in all the tissues, particularly the lungs (Fig 5D), but was low in plasma.

This was followed by a more detailed pharmacokinetic study, in which CD1 (n = 4; 8 weeks old, 26.8±3g) mice were administered a single dose of Up284, either IV (5 mg/kg), PO (50 mg/kg), or IP (20 mg/kg), and thereafter serial blood plasma samples were analyzed for Up284 levels by LC–MS/MS. The animals were randomly assigned to the treatment groups and fasted for 4 h before dosing (S13 Table). Nine time points for IV and IP (5, 15, 30, 60, 120, 240, 360, 480 and 1440 min) and eight time points for PO (15, 30, 60,120, 240, 360, 480 and 1440 min) administration were set for this pharmacokinetic study. Each of the time point treatment group included 4 animals. Mice were injected IP with 2,2,2-tribromoethanol (150 mg/kg) prior to drawing the blood in microtainers containing K_3_EDTA via retro-orbital bleeding. Blood samples were centrifuged 10 min at 3000 rpm. Brain samples (right lobe) were collected and weighted. All samples were immediately processed, flash-frozen and stored at -70°C until subsequent analysis. The average Up284 plasma concentrations data for IV (S14 Table), IP (S15 Table), and PO (S16 Table) dosed groups were analyzed graphically (Fig 5E). Pharmacokinetic parameters were calculated from mean concentration-time data using non-compartmental methods in Phoenix WinNonlin version 8.3 (Certara, Princeton, NJ)). Using the Method of Bailer, there was no different in dose normalized AUC_last_ by any route of administration (S17 Table). In summary, the calculated oral and IP bioavailability for Up284 was 23% and 87% respectively (S17 Table). No obvious adverse effects were observed during this PK study.

### Up284 treatment studies in mouse models of ovarian cancer

#### Orthotopic ES2-luc human ovarian cancer xenograft model

**Fig 6:**
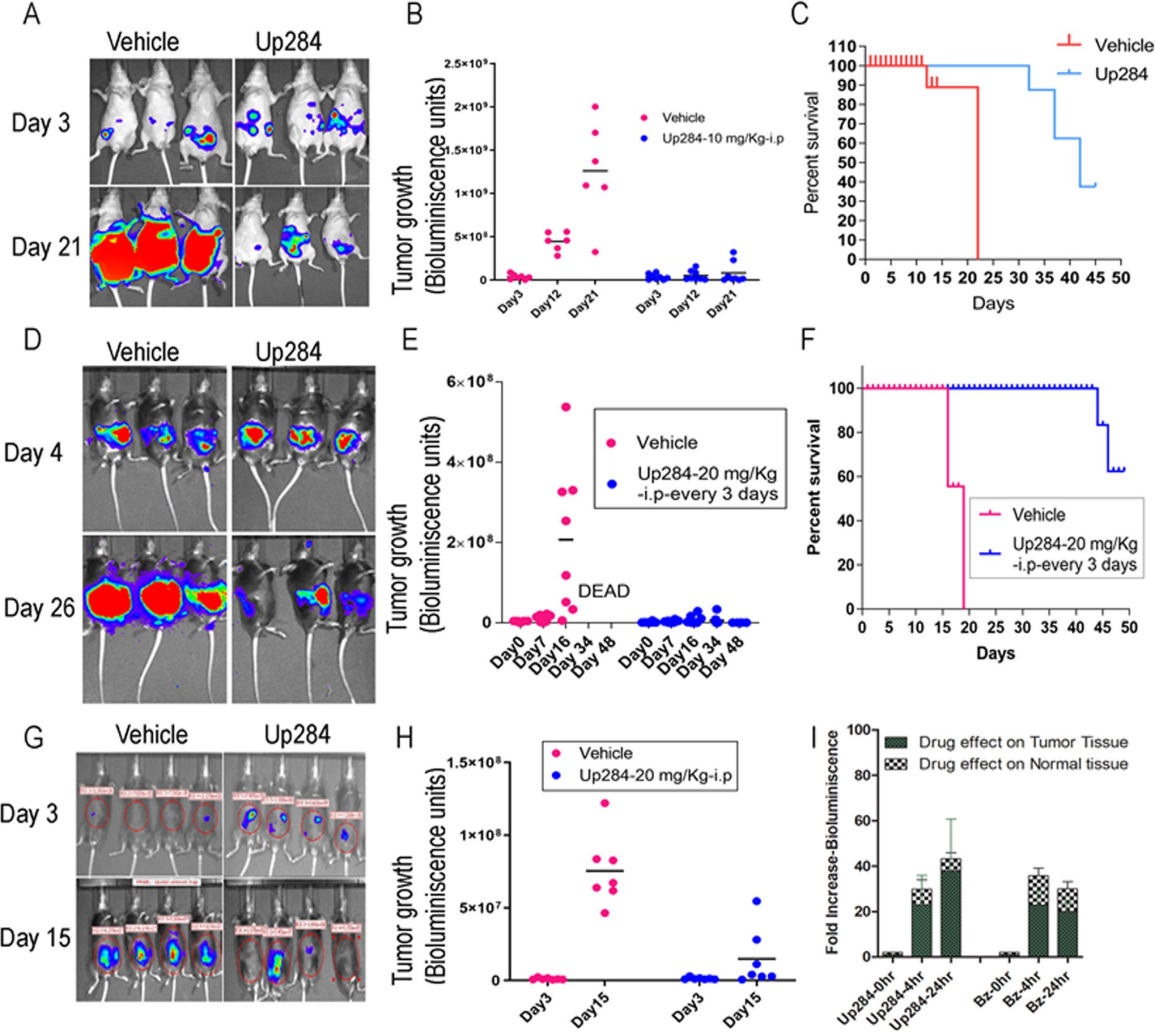
Impact of Up284 treatment upon tumor growth in xenograft, spontaneous and syngeneic mouse models of ovarian cancer. A-C) Female nude mice were inoculated IP with ES2-luc cells, a human ovarian cancer line expressing luciferase. Tumor growth was followed upon IP treatment with Up284 (n = 7, 10 mg/kg every 3 days for 2 weeks) or vehicle (25% β-hydroxypropyl cyclodextrin in water, n = 6) by IVIS imaging of bioluminescence (A-B), and survival was monitored (C); D-F) Female C57BL6 mice bearing spontaneous genetically engineered mouse model show tumor control with Up284 (n = 7, 20 mg/kg every 3 days delivered IP) as compared to vehicle treatment (n = 7) (D-E), and extended survival (F); G-H) Female C57BL6 mice bearing intraperitoneal syngeneic ID8-luc ovarian cancer model show tumor control with Up284 (n = 7, 20 mg/kg every 3 days, IP) as compared to vehicle treatment (n = 7). I) In vivo stabilization of 4UbFL reporter by Up284 (20 mg/kg, 1 dose delivered IP) or Bortezomib (0.75 mg/kg, 1 dose delivered IP) was followed by IVIS imaging in nude mice with established IP tumor derived from ES2 cells stably expressing 4UbFL (ES2-4UbFL) and concurrently in the leg muscle of the same animal electroporated with the 4UbFL reporter plasmid (n = 5). The IP tumor tissue and electroporated normal muscle tissue, being spatially distinct, were identified with separate regions of interest (ROI) after simultaneous imaging with IVIS.

The treatment efficacy of Up284 was first examined in female nude mice bearing ES2 human ovarian tumor expressing firefly luciferase (ES2-luc). Briefly mice were injected IP with ES2-luc cells, as ovarian cancer primarily spreads through the peritoneum, and it is the origin of primary peritoneal cancer. After four days, these mice were injected IP with luciferin and bioluminescence imaged by IVIS imager as a surrogate of tumor burden. Mice were randomized and treated IP with vehicle alone or 10 mg/kg Up284 every 3 days for 3 weeks. Bioluminescent imaging was performed by IVIS 12 and 21 days later. Treatment with Up284 significantly reduced ES2-luc xenograft tumor burden (p = 0.0002) compared to vehicle-treated mice at both time points and increased overall survival (p = 0.005) (Fig 6A–6C). Thus the Up284 treatment effect, presumably via direct tumor cell cytotoxicity, is evident in immunodeficient mice.

### ID8 syngeneic mouse model of ovarian cancer

Bortezomib and iRPN13 can trigger an immunogenic cell death promoted via cell surface display of calreticulin [16, 49]. Because of a potential role for antitumor immunity, the therapeutic effect of Up284 was tested against the syngeneic transplantable murine ovarian tumor model ID8-luc expressing firefly luciferase in C57BL6 with an intact immune system. Female C57BL6 mice (4–6 weeks) were challenged IP with ID8-luc tumor. Treatment was initiated 3 days later using a higher dose as ID8 is less sensitive to Up284 than ES2 (Fig 1B). The mice were administered IP either 20 mg/kg Up284 in 25% β-hydroxypropyl cyclodextrin in water, or vehicle alone, for 4 weeks every third day, and tumor growth was monitored using bioluminescent imaging. On day 15, the Up284-treated mice showed a significantly lower tumor burden (p = 0.0001) (Fig 6G and 6H). Furthermore, Up284 treatment significantly enhanced survival (p = 0.0004) in these immunocompetent mice.

### Spontaneous Genetically-Engineered Mouse Model (GEMM) of primary peritoneal/ovarian cancer

GEMM better mimic the spontaneous development of cancers in an immune competent host than transplantable tumor lines. Twenty female C57BL/6 mice were co-injected IP with sleeping beauty vector and plasmids expressing carrying shp53, AKT, c-Myc and firefly luciferase genes, followed by electroporation (EP) to deliver these plasmid DNA into the peritoneal cavity cells wherein they integrate randomly into their genome [32]. After 7 days mice were imaged by IVIS for bioluminescence and randomized. Groups (n = 10) were treated IP with vehicle or Up284 (20mg/kg) every 3 days for 6 weeks. Up284 significantly reduced tumor burden (p = 0.002) and showed a trend for prolonged survival compared to vehicle treated group (p = 0.06) (Fig 6D–6F). The vehicle treated group produced copious ascites and solid tumor, whereas the Up284 treated mice did not develop appreciable ascites and solid tumor burden was obviously lower upon dissection.

### Comparison of Up284 inhibition of proteasome function in healthy solid tissue and tumor

To investigate the Up284 activity in normal as compared to tumor tissue, a pilot experiment was performed in female nude mice. Briefly mice were inoculated IP with ES2 cells stably expressing 4UbFL. After 48 h, the mice were injected with luciferin, imaged and then randomized into 3 groups (n = 5/group). Next, leg muscle of each mouse was electroporated with 4UbFL plasmid (considered as normal tissue). After 48 h (i.e. day 4) the mice were re-imaged and basal bioluminescence (0 h) was separately measured in the leg muscle and peritoneal ROIs. The mice were then treated with a single IP dose of either vehicle, Up284 (20mg/kg) or bortezomib (0.75 mg/kg) and imaged again at 4 h and 24 h, and the bioluminescence signal normalized to that at 0 h for normal (leg) and tumor sites respectively. Up284 and bortezomib similarly enhanced the bioluminescence signal in muscle tissue, which trended higher than for tumor tissue, but did not reach significance (Fig 6I).

## Discussion

We developed a series of novel spirocyclic chemical scaffolds that bind to RPN13 with higher specificity than the prototype iRPN13, RA190, and other analogs in this bis-benzylidine piperidone series. Up284 appears most promising of this series, although Up285 is slightly more potent due to addition of another warhead, acryloyl, to the central spiro ring’s nitrogen. Use of nitrile electron withdrawing groups on the outer rings of the spirocyclic scaffold instead of nitro groups appears both to limit off-target effects due to the restriction of the spiro carbon atom, and to enhance potency [50, 51].

Up284 is more drug-like than RA190 and other analogs in this bis-benzylidine piperidone series as it lacks their PAINS structure and is more soluble. Up284 is well tolerated by mice at therapeutic doses with favorable PK/PD parameters when administered IP or IV, and it has potential even for oral administration. Although IP delivery of chemotherapy is most effective for ovarian cancer, it is less commonly used than IV administration [52]. When administered IP, Up284 provided significant treatment benefit against an orthotopic human ovarian cancer xenograft model, as well as syngeneic, and spontaneous genetically engineered mouse models. One possible reason that bortezomib is not effective for treatment of solid cancers is lack of access due to its peptide-based backbone. Indeed, more promising treatment effects were evident when bortezomib was administered IP to ovarian cancer patients [53] as compared to IV. Up284 has a very different scaffold. However, both agents effectively inhibited proteasome function in muscle and in ES2 ovarian cancer xenografts at 5 days post implantation (Fig 6I). This suggests that both Up284 and bortezomib readily access solid tumor. However, the tumor size is presumably small at only day 5 post-implantation as compared to much larger tumor volumes seen in patients, so differences in access to solid tumor might be less apparent in the mouse model.

Up284 demonstrated similar potency in bortezomib, cisplatin and paclitaxel resistant cell lines compared to parental lines cells (Fig 1B). Up284 also synergized with cisplatin in several ovarian cancer cell lines implying that Up284 may warrant testing as an approach to restore platinum-sensitivity in second line treatments. Importantly, Up284 was cytotoxic for cell lines from a variety of other cancer types, notably glioblastoma, MM, triple negative breast cancer, prostate, colon, and HPV-associated cervical cancers (Fig 1B), and efforts to validate these findings in animal models are ongoing.

As described with the bis-benzylidine piperidone series of candidate iRPN13, the potent cytotoxicity of Up284 is associated with both rapid accumulation of HMW polyUb proteins and mitochondrial dysfunction. This produces ER stress, drops in ATP levels and increased levels of ROS that trigger apoptosis [54]. A similar cytotoxic mechanism has been ascribed to bortezomib, and the emergence of resistance in myeloma has been linked to upregulation of antioxidant enzymes, notably SOD1. MM lines selected for resistance to bortezomib are similarly sensitive to RA190 (and Up284), and this likely reflects ability of RA190 to negate the upregulation of these antioxidant enzymes, including SOD1 [54]. Both bortezomib and iRPN13 is associated with mitochondrial membrane depolarization. Accumulation of ROS resulting from mitochondrial dysfunction can also trigger dissociation of 19S RP from the 20S CP, thereby inhibiting proteasome function [55], and this phenomenon is likewise evident upon treatment with either RA190 or bortezomib [54].

Despite many similarities, there are significant differences between in the mechanisms of action of bortezomib and iRPN13. The iRPN13, like Up284, produce an accumulation of very high MW polyUb proteins (barely running beyond the SDS-PAGE stacking gel) that is absent from cells treated with bortezomib. This suggests that adduction of RPN13 is preventing deubiquitination of the proteasome substrates, a prerequisite to their degradation. Inhibition of deubiquitination may occur by preventing substrate recognition by the 19S RP or/and prevention of RPN13 activation of the proteasomal deubiquitinases (DUB). The proteasomal DUB UCH37 is a logical candidate given its binding to and activation by RPN13, but it is not required for RA190-induced cytotoxicity [56]. A similar phenomenon is seen with b-AP15 and VLX1570 that are reported to inhibit the proteasomal DUBs UCH37 and USP14. However, Up284 did not broadly inhibit cellular DUBs or label proteins of molecular weight consistent with UCH37 or USP14 in tumor cell lysates. Thus, blockade of substrate recognition seems the most likely mechanism. Since bortezomib does not inhibit cellular DUB, this would potentially account for the higher MW of the polyUb protein accumulations seen in iRPN13-treated cells. It is also consistent with their more rapid killing of cells by iRPN13 than bortezomib. Cross-linking of the HMW polyUb protein accumulations by iRPN13 has been proposed, but no labelling was evident in this region in cancer cell lysates exposed to Up284B or RA190B arguing against this mechanism.

Like RA190 [19, 56–58], Up284B binds covalently to recombinant RPN13 *in vitro*. It also adducts a 42kDa cellular protein in cell lysates, a molecular weight consistent with RPN13. However more definitive studies are warranted to assign the target in live cells [59]. Importantly, evidence of proteasome inhibition by Up284 in muscle and tumor in live animals is provided by the stabilization of 4UbFL (Figs 5B and 5C and 6I). The inhibition of proteasome function in muscle of live mice lasts >48h from a single dose of Up284, suggesting dosing every third day is appropriate. Interestingly, Up284 clears from the bloodstream in 2–4 h to a low steady state and accumulates in major organs, notably lung, spleen liver and kidney at 24 h. We speculate that the extended period of 4UbFL stabilization *in vivo* by a single dose of Up284 may be due to the covalent drug binding as well as the time required by cells to synthesize new functional proteasomes (~24h), a reflection of their size and complexity.

While studies in nude mice show direct anticancer effects of RA190 on the ES2-luc xenograft model, our prior studies revealed that CD8 T cells contribute to its anticancer effect on the ID8-luc syngeneic model in C57BL6 mice [16]. This therapeutic effect was associated with RA190 inhibition MDSC-mediated suppression of CD8 T cell function. Such immunosuppressive mechanisms are particularly problematic in ovarian cancers and may account for the failures of immune checkpoint inhibitors so far in managing this disease. Here we show that RA190 and Up284 treatment can also enhance MHC class I presentation by tumor cells, albeit in a different C57BL6-derived cell line and using the model antigen OVA. This mechanism may contribute to the CD8 T cell dependent effects of RA190 in the ID8-luc model (as this line is relatively resistant to RA190 cytotoxicity as compared to ES2). Interestingly, bortezomib did not enhance antigen presentation in this model. This may reflect that bortezomib inhibits the immunoproteasomes responsible for cleaving proteins to peptides to enable their display by MHC class I. By contrast, the immunoproteasome uses PA28, which lacks RPN13, instead of 19S RP; therefore we speculate that iRPN13 would not inhibit peptide generation and thus antigen-presentation of SIINFEKL peptide derived from the accumulated OVA.

The induction of antitumor immunity upon bortezomib-induced immunogenic cell death (ICD) is a key component of its therapeutic efficacy against MM [49, 60]. RA190 similarly induces surface display of calreticulin, a hallmark of ICD, as for bortezomib. Low concentrations of RA190 promote activation of plasmacytoid dendritic cells derived from MM patients and thereby promote MM-specific CTLs and NK-cell-mediated cytotoxicity against autologous MM cells *in vitro* [61]. Taken together, these studies suggest the potential of this new iRPN13 Up284 to similarly abrogate immune suppression and restore antitumor immune responses, and the potential for combination with immune checkpoint inhibitors.

## Supporting information

S1 Fig Analysis of synergy of cisplatin and Up284 cytotoxicity for ovarian cancer cell lines. A) PEA2, a cell line developed from an ovarian cancer patient with cisplatin-resistant disease [62], was treated with Up284 and cisplatin for 72 h titrated in a checker board pattern in triplicate. Cell viability was measured using MTT assay and data was processed using the Synergy Finder web application. B) Same as in A, PACS cell line derived from spontaneous mouse ovarian tumor [32], was used.
(TIF)

S2 Fig Analysis of synergy of doxorubicin and Up284 cytotoxicity for ovarian cancer cell line SKOV3. SKOV3 cells were treated with Up284 and doxorubicin for 72 h titrated in a checker board pattern in triplicate. Cell viability was measured using MTT assay and data was processed using the Synergy Finder web application.
(TIF)

S1 Raw images Unmodified images of all blot analyses showing whole gel. (PDF)

S1 Table Stability of Up284 in murine and human plasma. (DOCX)

S2 Table Mouse plasma protein binding. (DOCX)

S3 Table Stability of Up284 in murine and human liver microsomes with or without exogenous NADPH. (DOCX)

S4 Table Design of single administration dose escalation study for Up284 in female CD1 mice (9 weeks old). (DOCX)

S5 Table Body weights in single administration dose escalation study for Up284 in female CD1 mice (9 weeks old). (DOCX)

S6 Table Clinical observations in single administration dose escalation study for Up284 in female CD1 mice (9 weeks old). (DOCX)

S7 Table Hematological observations in single administration dose escalation study for Up284 in female CD1 mice (9 weeks old). (DOCX)

S8 Table Repeat dose toxicity study for Up284 and bortezomib (IV in 5 mL/kg) in female CD1 mice (9 weeks old). (DOCX)

S9 Table Body weights in repeat dose toxicity study for Up284 and bortezomib in female CD1 mice (9 weeks old). (DOCX)

S10 Table Clinical observations in repeat dose toxicity study for Up284 and bortezomib in female CD1 mice (9 weeks old). (DOCX)

S11 Table Hematologic parameters in repeat dose toxicity study for Up284 and bortezomib in female CD1 mice (9 weeks old). (DOCX)

S12 Table Blood chemistry parameters in repeat dose toxicity study for Up284 and bortezomib in female CD1 mice (9 weeks old). (DOCX)

S13 Table Study to determine the pharmacokinetic characteristics of compound Up284 in male CD1 mice (8 weeks old) following intravenous (IV), intraperitoneal (IP) and per oral (PO) administration. (DOCX)

S14 Table Plasma concentrations of Up284 in male CD1 mice following IV (5 mg/kg) administration. (DOCX)

S15 Table Plasma concentrations for Up284 in CD-1 mice following IP (20 mg/kg) administration. (DOCX)

S16 Table Plasma concentrations of Up284 in CD-1 mice following PO (50 mg/kg) administration. (DOCX)

S17 Table Comparison of selected pharmacokinetic parameters for Up284 in male CD1 mice. (DOCX)

S1 File Synthesis of candidate iRPN13. Description of chemical synthesis of compounds in Table 1.
(DOCX)
