# Supplementary figures and images for: Development and anticancer properties of Up284, a spirocyclic candidate ADRM1/RPN13 inhibitor
Source: PLoS One. 2023 Jun 14;18(6):e0285221. doi: 10.1371/journal.pone.0285221 (PMC10266688; doi:10.1371/journal.pone.0285221)

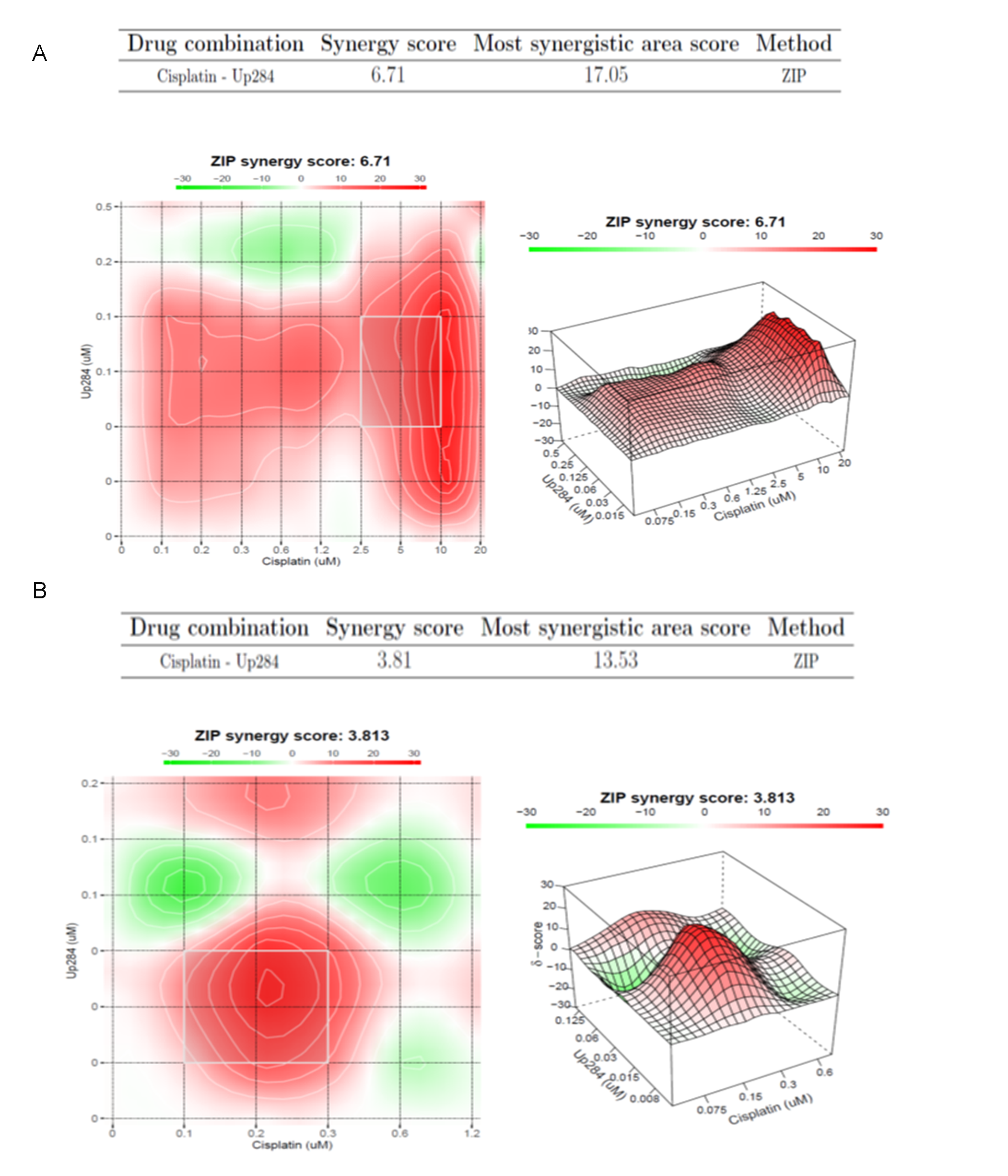

Supplement: S1 Fig — A) PEA2, a cell line developed from an ovarian cancer patient with cisplatin-resistant disease [62], was treated with Up284 and cisplatin for 72 h titrated in a checker board pattern in triplicate. Cell viability was measured using MTT assay and data was processed using the Synergy Finder web application. B) Same as in A, PACS cell line derived from spontaneous mouse ovarian tumor [32], was used. (TIF) [file pone.0285221.s001.tif]

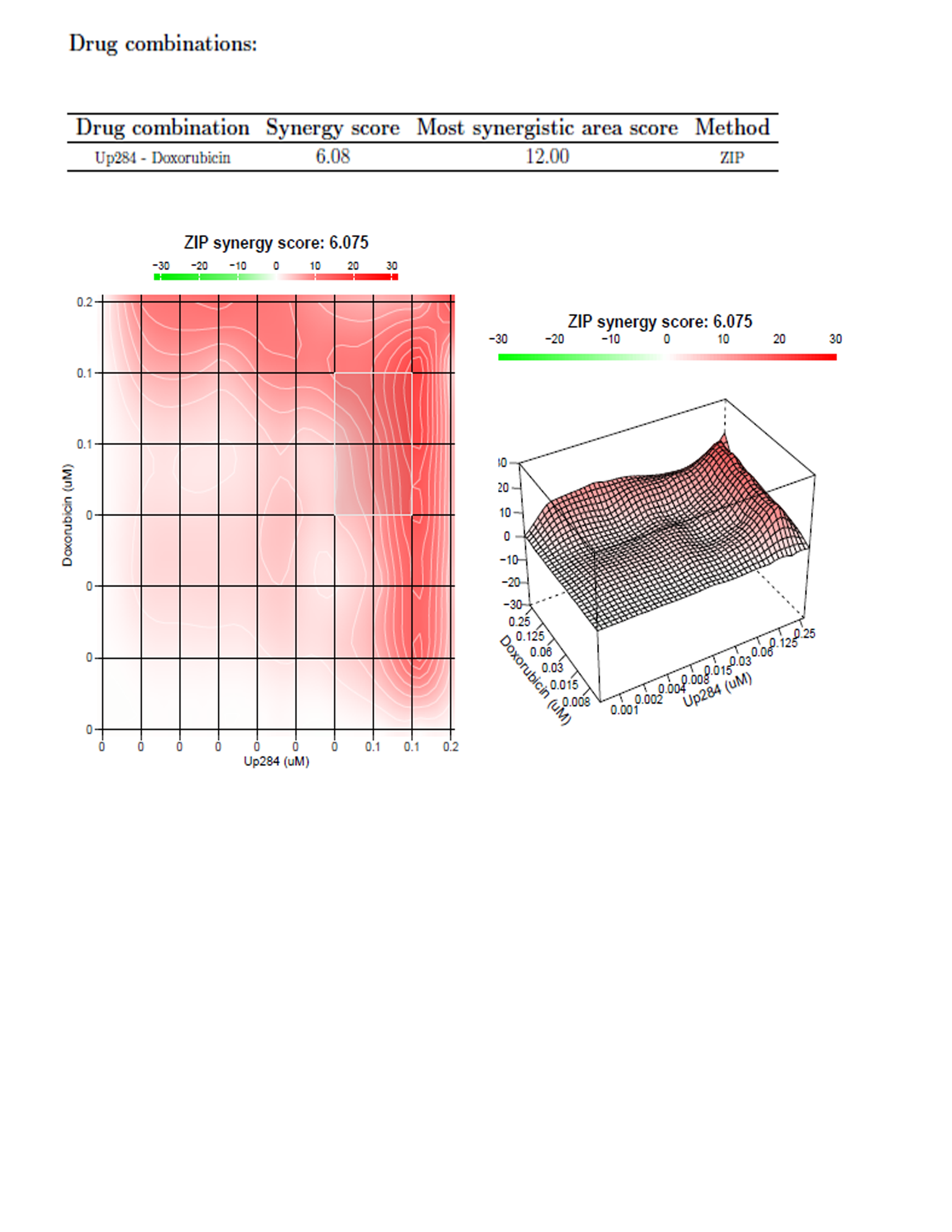

Supplement: S2 Fig — SKOV3 cells were treated with Up284 and doxorubicin for 72 h titrated in a checker board pattern in triplicate. Cell viability was measured using MTT assay and data was processed using the Synergy Finder web application. (TIF) [file pone.0285221.s002.tif]
