# Supplementary material for: Development and anticancer properties of Up284, a spirocyclic candidate ADRM1/RPN13 inhibitor
Source: PLoS One. 2023 Jun 14;18(6):e0285221. doi: 10.1371/journal.pone.0285221 (PMC10266688; doi:10.1371/journal.pone.0285221)
Supplement: S1 Table — (DOCX) [file pone.0285221.s004.docx]

Table S1. Stability of Up284 in murine and human plasma.

| Stability Matrix | Neat Solution | Plasma (Mouse EDTA) | Plasma (Human EDTA) |
| --- | --- | --- | --- |
| Time (minutes) | % Drug Remaining | % Drug Remaining | % Drug Remaining |
| 0 | 100% | 100% | 100% |
| 30 | 86% | 84% | 100% |
| 60 | 78% | 94% | 90% |
| 60 | 78% | 94% | 90% |
