# Supplementary material for: Development and anticancer properties of Up284, a spirocyclic candidate ADRM1/RPN13 inhibitor
Source: PLoS One. 2023 Jun 14;18(6):e0285221. doi: 10.1371/journal.pone.0285221 (PMC10266688; doi:10.1371/journal.pone.0285221)
Supplement: S2 Table — (DOCX) [file pone.0285221.s005.docx]

Table S2. Mouse plasma protein binding.

| **Compound ID** | **Concentration, µM** | **Incubation #** | **Peak area ratio** | | **% of bound compound** | **% of bound compound, mean** | **Recovery, %** | **Stability,**  **%** |
| --- | --- | --- | --- | --- | --- | --- | --- | --- |
|  |  |  | **Buffer** | **Plasma** |  |  |  |  |
| Verapamil | 1.0 | # 1 | 2.25E-03 | 2.78E-02 | 92 | **92** | 99 | 107 |
|  |  | # 2 | 2.34E-03 | 2.84E-02 | 92 |  | 102 |  |
| **Up284** | 1.0 | # 1 | 1.40E-03 | 6.85E-02 | 98 | **98** | 98 | 109 |
|  |  | # 2 | 1.77E-03 | 6.97E-02 | 97 |  | 101 |  |
