# Supplementary material for: Development and anticancer properties of Up284, a spirocyclic candidate ADRM1/RPN13 inhibitor
Source: PLoS One. 2023 Jun 14;18(6):e0285221. doi: 10.1371/journal.pone.0285221 (PMC10266688; doi:10.1371/journal.pone.0285221)
Supplement: S3 Table — (DOCX) [file pone.0285221.s006.docx]

Table S3. Stability of Up284 in murine and human liver microsomes with or without exogenous NADPH.

| Stability Matrix | Mouse Liver Microsomes | | Human Liver Microsomes | |
| --- | --- | --- | --- | --- |
| Time (minutes) | % Drug Remaining NADPH Neg | % Drug Remaining  NADPH Pos | % Drug Remaining NADPH Neg | % Drug Remaining NADPH Pos |
| 0 | 100% | 100% | 100% | 100% |
| 30 | 60% | 45% | 57% | 43% |
| 60 | 51% | 38% | 52% | 43% |
