# Supplementary material for: Development and anticancer properties of Up284, a spirocyclic candidate ADRM1/RPN13 inhibitor
Source: PLoS One. 2023 Jun 14;18(6):e0285221. doi: 10.1371/journal.pone.0285221 (PMC10266688; doi:10.1371/journal.pone.0285221)
Supplement: S4 Table — (DOCX) [file pone.0285221.s007.docx]

Table S4. Design of single administration dose escalation study for Up284 in female CD1 mice (9 weeks old).

| Day # | IV | | | IP | | | | | PO | | |
| --- | --- | --- | --- | --- | --- | --- | --- | --- | --- | --- | --- |
|  | Group 1 | Group 2 | Group 3 | Group 4 | Group 5 | Group 6 | Group 7 | Group 8 | Group 9 | Group 10 | Group 11 |
|  | Vehicle | Up284, 40 mg/kg | Up284, 60 mg/kg | Vehicle | Up284, 40 mg/kg | Up284, 60 mg/kg | Up284, 80 mg/kg | Up284, 100 mg/kg | Vehicle | Up284, 100 mg/kg | Up284, 200 mg/kg |
|  | Number of mice: 3 | 3 | 3 | 3 | 3 | 3 | 3 | 3 | 3 | 3 | 3 |
| 1 | X Gc W | X Gc W |  | X Gc W | X Gc W |  |  |  |  |  |  |
| 2 | Gc W | Gc W | X Gc W | Gc W | Gc W | X Gc W |  |  | X Gc W | X Gc W |  |
| 3 | Gc W | Gc W | Gc W | Gc W | Gc W | Gc W | X Gc W |  | Gc W | Gc W | X Gc W |
| 4 | Gc W | Gc W | Gc W | Gc W | Gc W | Gc W | Gc W | X Gc W | Gc W | Gc W | Gc W |
| 5 | Gc W | Gc W | Gc W | Gc W | Gc W | Gc W | Gc W | Gc W | Gc W | Gc W | Gc W |
| 6 | Gc W | Gc W | Gc W | Gc W | Gc W | Gc W | Gc W | Gc W | Gc W | Gc W | Gc W |
| 7 | Gc W | Gc W | Gc W | Gc W | Gc W | Gc W | Gc W | Gc W | Gc W | Gc W | Gc W |
| 8 | Bt Gc W | Gc W | Gc W | Bt Gc W | Bt Gc W | Gc W | Gc W | Gc W | Gc W | Gc W | Gc W |
| 9 |  |  | Bt Gc W |  |  | Gc W | Gc W | Gc W | Bt Gc W | Gc W | Gc W |
| 10 |  |  |  |  |  |  | Gc W | Gc W |  |  | Bt Gc W |
| 11 |  |  |  |  |  |  |  | Bt Gc W |  |  |  |

Bt - terminal bleeding of survived mice with the maximum tested doses for hematology and clinical chemistry (ALAT, ASAT, LDH)

Gc - general condition observation

W – weighing

X - treatment with compound or vehicle
