# Supplementary material for: Development and anticancer properties of Up284, a spirocyclic candidate ADRM1/RPN13 inhibitor
Source: PLoS One. 2023 Jun 14;18(6):e0285221. doi: 10.1371/journal.pone.0285221 (PMC10266688; doi:10.1371/journal.pone.0285221)
Supplement: S5 Table — (DOCX) [file pone.0285221.s008.docx]

Table S5. Body weights in single administration dose escalation study for Up284 in female CD1 mice (9 weeks old) by IV, IP or PO.

| **IV route** | | | | | | | | | | | | |
| --- | --- | --- | --- | --- | --- | --- | --- | --- | --- | --- | --- | --- |
| Cage # | Mouse # | Compound, dose | Body weight at different days of the study, g | | | | | | | | Body weight change*, % | |
|  |  |  | 0 | 1 | 2 | 3 | 4 | 5 | 6 | 7 |  |  |
| 16 | 16 | Vehicle | 23.4 | 23.4 | 23.5 | 23.8 | 23.6 | 24.2 | 23.8 | 24.1 | 2.99 |  |
| 23 | 23 |  | 26.6 | 25.7 | 25.9 | 26.5 | 26.7 | 27.3 | 26.8 | 26.4 | -0.75 |  |
| 33 | 33 |  | 21.6 | 20.5 | 20.5 | 21.3 | 21.8 | 22.1 | 22.3 | 21.9 | 1.39 |  |
| Mean | | | **23.87** | **23.20** | **23.30** | **23.87** | **24.03** | **24.53** | **24.30** | **24.13** | **1.21** |  |
| SD | | | **2.53** | **2.61** | **2.71** | **2.60** | **2.48** | **2.62** | **2.29** | **2.25** | **1.88** |  |
| SE | | | **1.46** | **1.50** | **1.56** | **1.50** | **1.43** | **1.51** | **1.32** | **1.30** | **1.08** |  |
| 2 | 2 | Up284, 40 mg/kg | 26.2 | 25.5 | 25.7 | 26.5 | 27.7 | 28.0 | 27.2 | 27.2 | 3.82 |  |
| 12 | 12 |  | 23.2 | 22.6 | 22.4 | 23.6 | 22.9 | 23.0 | 22.6 | 23.2 | 0.00 |  |
| 21 | 21 |  | 24.1 | 23.6 | 24.1 | 25.0 | 25.1 | 24.5 | 25.1 | 24.8 | 2.90 |  |
| Mean | | | **24.50** | **23.90** | **24.07** | **25.03** | **25.23** | **25.17** | **24.97** | **25.07** | **2.24** |  |
| SD | | | **1.54** | **1.47** | **1.65** | **1.45** | **2.40** | **2.57** | **2.30** | **2.01** | **1.99** |  |
| SE | | | **0.89** | **0.85** | **0.95** | **0.84** | **1.39** | **1.48** | **1.33** | **1.16** | **1.15** |  |
| **P (t-test, compared to Vehicle)** | | | 0.7300 | 0.7062 | 0.6967 | 0.5346 | 0.5796 | 0.7796 | 0.7402 | 0.6208 | 0.5499 |  |
| 3 | 3 | Up284, 60 mg/kg | 26.0 | 26.7 | 27.1 | 26.5 | 26.6 | 26.5 | 26.7 | 26.6 | 2.31 |  |
| 24 | 24 |  | 22.7 | 22.6 | 23.4 | 23.7 | 23.5 | 23.3 | 24.1 | 23.9 | 5.29 |  |
| 31 | 31 |  | 24.6 | 24.1 | 23.8 | 24.0 | 24.7 | 24.7 | 25.1 | 25.3 | 2.85 |  |
| Mean | | | **24.43** | **24.47** | **24.77** | **24.73** | **24.93** | **24.83** | **25.30** | **25.27** | **3.48** |  |
| SD | | | **1.66** | **2.07** | **2.03** | **1.54** | **1.56** | **1.60** | **1.31** | **1.35** | **1.59** |  |
| SE | | | **0.96** | **1.20** | **1.17** | **0.89** | **0.90** | **0.93** | **0.76** | **0.78** | **0.92** |  |
| **P (t-test, compared to Vehicle)** | | | 0.7619 | 0.5461 | 0.4944 | 0.6453 | 0.6229 | 0.8738 | 0.5476 | 0.4960 | 0.1850 |  |

| **IP route** | | | | | | | | | | | |
| --- | --- | --- | --- | --- | --- | --- | --- | --- | --- | --- | --- |
| Cage # | Mouse # | Compound, dose | Body weight at different days of the study, g | | | | | | | | Body weight change*, % |
|  |  |  | 0 | 1 | 2 | 3 | 4 | 5 | 6 | 7 |  |
| 14 | 14 | Vehicle | 22.1 | 21.1 | 21.2 | 22.0 | 22.3 | 22.7 | 22.3 | 21.9 | -0.90 |
| 25 | 25 |  | 22.5 | 23.1 | 23.3 | 23.4 | 22.7 | 23.1 | 23.2 | 24.3 | 8.00 |
| 36 | 36 |  | 27.3 | 26.3 | 26.8 | 27.1 | 28.0 | 28.5 | 27.8 | 27.2 | -0.37 |
| Mean | | | **23.97** | **23.50** | **23.77** | **24.17** | **24.33** | **24.77** | **24.43** | **24.47** | **2.24** |
| SD | | | **2.89** | **2.62** | **2.83** | **2.64** | **3.18** | **3.24** | **2.95** | **2.65** | **4.99** |
| SE | | | **1.67** | **1.51** | **1.63** | **1.52** | **1.84** | **1.87** | **1.70** | **1.53** | **2.88** |
| 4 | 4 | Up284, 40 mg/kg | 25.6 | 23.9 | 23.3 | 24.2 | 24.5 | 25.0 | 25.3 | 26.2 | 2.34 |
| 18 | 18 |  | 24.3 | 22.7 | 23.3 | 24.5 | 24.6 | 25.1 | 24.6 | 24.5 | 0.82 |
| 22 | 22 |  | 22.7 | 21.9 | 22.3 | 22.7 | 22.5 | 23.2 | 22.5 | 23.8 | 4.85 |
| Mean | | | **24.20** | **22.83** | **22.97** | **23.80** | **23.87** | **24.43** | **24.13** | **24.83** | **2.67** |
| SD | | | **1.45** | **1.01** | **0.58** | **0.96** | **1.18** | **1.07** | **1.46** | **1.23** | **2.03** |
| SE | | | **0.84** | **0.58** | **0.33** | **0.56** | **0.68** | **0.62** | **0.84** | **0.71** | **1.17** |
| **P (t-test, compared to Vehicle)** | | | 0.9067 | 0.7021 | 0.6564 | 0.8320 | 0.8235 | 0.8738 | 0.8822 | 0.8388 | 0.8973 |
| 1 | 1 | Up284, 60 mg/kg | 24.4 | died |  |  |  |  |  |  | -100.00 |
| 28 | 28 |  | 25.1 | died |  |  |  |  |  |  | -100.00 |
| 32 | 32 |  | 22.5 | died |  |  |  |  |  |  | -100.00 |
| Mean | | | **24.00** | **######** | **######** | **######** | **######** | **######** | **######** | **######** | **-100.00** |
| SD | | | **1.35** | **######** | **######** | **######** | **######** | **######** | **######** | **######** | **0.00** |
| SE | | | **0.78** | **######** | **######** | **######** | **######** | **######** | **######** | **######** | **0.00** |
| **P (t-test, compared to Vehicle)** | | | 0.9864 | ###### | ###### | ###### | ###### | ###### | ###### | ###### | 0.0000 |

| **PO route** | | | | | | | | | | | |
| --- | --- | --- | --- | --- | --- | --- | --- | --- | --- | --- | --- |
| Cage # | Mouse # | Compound, dose | Body weight at different days of the study, g | | | | | | | | Body weight change*, % |
|  |  |  | 0 | 1 | 2 | 3 | 4 | 5 | 6 | 7 |  |
| 10 | 10 | Vehicle | 21.3 | 21.7 | 21.8 | 22.4 | 22.1 | 21.8 | 21.4 | 22.4 | 5.16 |
| 20 | 20 |  | 23.6 | 23.7 | 24.3 | 25.1 | 24.5 | 23.8 | 23.7 | 24.1 | 2.12 |
| 30 | 30 |  | 27.3 | 27.3 | 27.0 | 27.7 | 27.3 | 28.2 | 28.5 | 28.5 | 4.40 |
| Mean | | | **24.07** | **24.23** | **24.37** | **25.07** | **24.63** | **24.60** | **24.53** | **25.00** | **3.89** |
| SD | | | **3.03** | **2.84** | **2.60** | **2.65** | **2.60** | **3.27** | **3.62** | **3.15** | **1.58** |
| SE | | | **1.75** | **1.64** | **1.50** | **1.53** | **1.50** | **1.89** | **2.09** | **1.82** | **0.91** |
| 11 | 11 | Up284, 100 mg/kg | 27.5 | 26.4 | 27.0 | 27.2 | 27.9 | 26.9 | 28.4 | 29.3 | 6.55 |
| 17 | 17 |  | 24.7 | 24.4 | 24.5 | 25.0 | 24.5 | 24.4 | 25.0 | 25.6 | 3.64 |
| 27 | 27 |  | 21.9 | 22.1 | 22.8 | 23.1 | 23.5 | 23.0 | 23.1 | 23.4 | 6.85 |
| Mean | | | **24.70** | **24.30** | **24.77** | **25.10** | **25.30** | **24.77** | **25.50** | **26.10** | **5.68** |
| SD | | | **2.80** | **2.15** | **2.11** | **2.05** | **2.31** | **1.98** | **2.69** | **2.98** | **1.77** |
| SE | | | **1.62** | **1.24** | **1.22** | **1.18** | **1.33** | **1.14** | **1.55** | **1.72** | **1.02** |
| **P (t-test, compared to Vehicle)** | | | 0.8034 | 0.9757 | 0.8463 | 0.9871 | 0.7565 | 0.9435 | 0.7292 | 0.6831 | 0.2625 |
| 5 | 5 | Up284, 200 mg/kg | 24.2 | 24.9 | 25.7 | 25.6 | 25.1 | 25.1 | 25.8 | 26.3 | 8.68 |
| 34 | 34 |  | 26.3 | 25.8 | 25.5 | 25.1 | 26.3 | 26.2 | 25.7 | 26.3 | 0.00 |
| 37 | 37 |  | 23.6 | 24.0 | 24.3 | 24.5 | 24.8 | 24.7 | 25.4 | 25.0 | 5.93 |
| Mean | | | **24.70** | **24.90** | **25.17** | **25.07** | **25.40** | **25.33** | **25.63** | **25.87** | **4.87** |
| SD | | | **1.42** | **0.90** | **0.76** | **0.55** | **0.79** | **0.78** | **0.21** | **0.75** | **4.44** |
| SE | | | **0.82** | **0.52** | **0.44** | **0.32** | **0.46** | **0.45** | **0.12** | **0.43** | **2.56** |
| **P (t-test, compared to Vehicle)** | | | 0.7592 | 0.7179 | 0.6359 | 1.0000 | 0.6511 | 0.7250 | 0.6273 | 0.6669 | 0.7375 |

* Body weight change compared to the initial weight was calculated for the 7th day of the study
