# Supplementary material for: Development and anticancer properties of Up284, a spirocyclic candidate ADRM1/RPN13 inhibitor
Source: PLoS One. 2023 Jun 14;18(6):e0285221. doi: 10.1371/journal.pone.0285221 (PMC10266688; doi:10.1371/journal.pone.0285221)
Supplement: S6 Table — (DOCX) [file pone.0285221.s009.docx]

Table S6. Clinical observations in single administration dose escalation study for Up284 in female CD1 mice (9 weeks old)

Key: 0 – good appearance, D - hypokinesia (decreased activity), Eh - half-closed eyes, H - hunched posture, P - piloerection

| Cage # | Mouse # | Route | Compound, dose | Days and hours of observations | | | | | | | | | | | |
| --- | --- | --- | --- | --- | --- | --- | --- | --- | --- | --- | --- | --- | --- | --- | --- |
|  |  |  |  | 0 | | | | | 1 | 2 | 3 | 4 | 5 | 6 | 7 |
|  |  |  |  | 0 | 0.5 h | 2 h | 4 h | 6 h |  |  |  |  |  |  |  |
| 16 | 16 | IV | Vehicle | 0 | 0 | 0 | 0 | 0 | 0 | 0 | 0 | 0 | 0 | 0 | 0 |
| 23 | 23 |  |  | 0 | 0 | 0 | 0 | 0 | 0 | 0 | 0 | 0 | 0 | 0 | 0 |
| 33 | 33 |  |  | 0 | 0 | 0 | 0 | 0 | 0 | 0 | 0 | 0 | 0 | 0 | 0 |
| 2 | 2 |  | Up284, 40 mg/kg | 0 | Eh | 0 | 0 | 0 | 0 | 0 | 0 | 0 | 0 | 0 | 0 |
| 12 | 12 |  |  | 0 | Eh | 0 | 0 | 0 | 0 | 0 | 0 | 0 | 0 | 0 | 0 |
| 21 | 21 |  |  | 0 | Eh | 0 | 0 | 0 | 0 | 0 | 0 | 0 | 0 | 0 | 0 |
| 3 | 3 |  | Up284, 60 mg/kg | 0 | P | 0 | 0 | 0 | 0 | 0 | 0 | 0 | 0 | 0 | 0 |
| 24 | 24 |  |  | 0 | 0 | 0 | 0 | 0 | 0 | 0 | 0 | 0 | 0 | 0 | 0 |
| 31 | 31 |  |  | 0 | 0 | 0 | 0 | 0 | 0 | 0 | 0 | 0 | 0 | 0 | 0 |
| 14 | 14 | IP | Vehicle | 0 | 0 | 0 | 0 | 0 | 0 | 0 | 0 | 0 | 0 | 0 | 0 |
| 25 | 25 |  |  | 0 | 0 | 0 | 0 | 0 | 0 | 0 | 0 | 0 | 0 | 0 | 0 |
| 36 | 36 |  |  | 0 | 0 | 0 | 0 | 0 | 0 | 0 | 0 | 0 | 0 | 0 | 0 |
| 4 | 4 |  | Up284, 40 mg/kg | 0 | DPEh | HDPEh | HDPEh | DPEh | P | 0 | 0 | 0 | 0 | 0 | 0 |
| 18 | 18 |  |  | 0 | DPEh | HDPEh | HDPEh | DPEh | 0 | 0 | 0 | 0 | 0 | 0 | 0 |
| 22 | 22 |  |  | 0 | DPEh | HDPEh | HDPEh | DPEh | P | 0 | 0 | 0 | 0 | 0 | 0 |
| 1 | 1 |  | Up284, 60 mg/kg | 0 | DPEh | HDPEh | HDPEh | DPEh | died |  |  |  |  |  |  |
| 28 | 28 |  |  | 0 | DPEh | HDPEh | HDPEh | DPEh | died |  |  |  |  |  |  |
| 32 | 32 |  |  | 0 | DPEh | HDPEh | HDPEh | DPEh | died |  |  |  |  |  |  |
| 10 | 10 | PO | Vehicle | 0 | 0 | 0 | 0 | 0 | 0 | 0 | 0 | 0 | 0 | 0 | 0 |
| 20 | 20 |  |  | 0 | 0 | 0 | 0 | 0 | 0 | 0 | 0 | 0 | 0 | 0 | 0 |
| 30 | 30 |  |  | 0 | 0 | 0 | 0 | 0 | 0 | 0 | 0 | 0 | 0 | 0 | 0 |
| 11 | 11 |  | Up284, 100 mg/kg | 0 | 0 | 0 | 0 | 0 | 0 | 0 | 0 | 0 | 0 | 0 | 0 |
| 17 | 17 |  |  | 0 | 0 | 0 | 0 | 0 | 0 | 0 | 0 | 0 | 0 | 0 | 0 |
| 27 | 27 |  |  | 0 | 0 | 0 | 0 | 0 | 0 | 0 | 0 | 0 | 0 | 0 | 0 |
| 5 | 5 |  | Up284, 200 mg/kg | 0 | 0 | 0 | 0 | 0 | 0 | 0 | 0 | 0 | 0 | 0 | 0 |
| 34 | 34 |  |  | 0 | 0 | 0 | 0 | 0 | 0 | 0 | 0 | 0 | 0 | 0 | 0 |
| 37 | 37 |  |  | 0 | 0 | 0 | 0 | 0 | 0 | 0 | 0 | 0 | 0 | 0 | 0 |
