# Supplementary material for: Development and anticancer properties of Up284, a spirocyclic candidate ADRM1/RPN13 inhibitor
Source: PLoS One. 2023 Jun 14;18(6):e0285221. doi: 10.1371/journal.pone.0285221 (PMC10266688; doi:10.1371/journal.pone.0285221)
Supplement: S7 Table — (DOCX) [file pone.0285221.s010.docx]

Table S7. Hematological observations in single administration dose escalation study for Up284 in female CD1 mice (9 weeks old)

Final blood parameters values IV

| Cage # | mouse # | Compound, dose | WBC, 10^9^/L | LYM, % | MID, % | GRAN, % | LYM, 10^9^/L | MID, 10^9^/L | GRAN, 10^9^/L | RBC, 10^12^/L | HGB, g/L | HCT, % | MCV, fL | MCH, pg | MCHC, g/L | RDW-SD, fL | RDW-CV, % | PLT, 10^9^/L | MPV, fL |
| --- | --- | --- | --- | --- | --- | --- | --- | --- | --- | --- | --- | --- | --- | --- | --- | --- | --- | --- | --- |
| 16 | 16 | Vehicle IV | 4.40 | 57.3 | 7.8 | 34.9 | 2.52 | 0.34 | 1.54 | 9.78 | 172 | 48.20 | 49.40 | 17.50 | 355 | 17.70 | 14.30 | 928 | 5.30 |
| 23 | 23 |  | 4.20 | 47.1 | 8.2 | 44.7 | 1.98 | 0.34 | 1.88 | 10.14 | 186 | 49.00 | 48.30 | 18.30 | 378 | 18.00 | 14.90 | 1070 | 5.70 |
| 33 | 33 |  | 3.20 | 68.6 | 5.8 | 25.6 | 2.20 | 0.19 | 0.82 | 9.50 | 180 | 47.80 | 50.30 | 18.90 | 376 | 18.80 | 14.90 | 1084 | 5.50 |
| Mean | | | **3.93** | **57.67** | **7.27** | **35.07** | **2.23** | **0.29** | **1.41** | **9.81** | **179** | **48.33** | **49.33** | **18.23** | **370** | **18.17** | **14.70** | **1027** | **5.50** |
| SD | | | **0.64** | **10.75** | **1.29** | **9.55** | **0.27** | **0.09** | **0.54** | **0.32** | **7.02** | **0.61** | **1.00** | **0.70** | **12.74** | **0.57** | **0.35** | **86.31** | **0.20** |
| SE | | | **0.37** | **6.21** | **0.74** | **5.51** | **0.16** | **0.05** | **0.31** | **0.19** | **4.06** | **0.35** | **0.58** | **0.41** | **7.36** | **0.33** | **0.20** | **49.83** | **0.12** |
| 3 | 3 | Up284, 60 mg/kg IV | 2.20 | 56.2 | 4.9 | 38.9 | 1.24 | 0.11 | 0.86 | 9.66 | 178 | 47.20 | 48.90 | 18.40 | 376 | 18.30 | 14.90 | 854 | 5.50 |
| 24 | 24 |  | 4.00 | 63.9 | 7.9 | 28.2 | 2.56 | 0.32 | 1.13 | 9.64 | 174 | 46.60 | 48.50 | 18.10 | 374 | 18.30 | 15.10 | 962 | 5.30 |
| 31 | 31 |  | 2.00 | 57.6 | 8.3 | 34.1 | 1.15 | 0.17 | 0.68 | 9.40 | 170 | 46.40 | 49.50 | 18.10 | 366 | 18.20 | 14.70 | 1142 | 5.50 |
| Mean | | | **2.73** | **59.23** | **7.03** | **33.73** | **1.65** | **0.20** | **0.89** | **9.57** | **174** | **46.73** | **48.97** | **18.20** | **372** | **18.27** | **14.90** | **986** | **5.43** |
| SD | | | **1.10** | **4.10** | **1.86** | **5.36** | **0.79** | **0.11** | **0.22** | **0.14** | **4.00** | **0.42** | **0.50** | **0.17** | **5.29** | **0.06** | **0.20** | **145.49** | **0.12** |
| SE | | | **0.64** | **2.37** | **1.07** | **3.09** | **0.45** | **0.06** | **0.13** | **0.08** | **2.31** | **0.24** | **0.29** | **0.10** | **3.06** | **0.03** | **0.12** | **84.00** | **0.07** |
| **P (t-test, compared to Vehicle)** | | | 0.1785 | 0.8252 | 0.8668 | 0.8433 | 0.2921 | 0.3104 | 0.1970 | 0.3030 | 0.3169 | 0.0200 | 0.6013 | 0.9402 | 0.7841 | 0.7770 | 0.4353 | 0.6939 | 0.6433 |

Final blood parameters values IP

| Cage # | mouse # | Compound, dose | WBC, 10^9^/L | LYM, % | MID, % | GRAN, % | LYM, 109/L | MID, 109/L | GRAN, 109/L | RBC, 10^12^/L | HGB, g/L | HCT, % | MCV, fL | MCH, pg | MCHC, g/L | RDW-SD, fL | RDW-CV, % | PLT, 10^9^/L | MPV, fL |
| --- | --- | --- | --- | --- | --- | --- | --- | --- | --- | --- | --- | --- | --- | --- | --- | --- | --- | --- | --- |
| 14 | 14 | Vehicle IP | 4.80 | 58.5 | 7.4 | 34.1 | 2.81 | 0.36 | 1.64 | 9.00 | 166 | 44.40 | 49.30 | 18.50 | 375 | 18.10 | 14.70 | 1130 | 5.50 |
| 25 | 25 |  | 2.60 | 62.6 | 4.3 | 33.1 | 1.63 | 0.11 | 0.86 | 9.54 | 174 | 46.00 | 48.20 | 18.30 | 379 | 18.00 | 15.00 | 864 | 5.70 |
| 36 | 36 |  | 2.60 | 58.3 | 8.3 | 33.4 | 1.52 | 0.22 | 0.87 | 9.56 | 166 | 45.80 | 48.00 | 17.40 | 362 | 17.70 | 14.80 | 874 | 5.40 |
| Mean | | | **3.33** | **59.80** | **6.67** | **33.53** | **1.98** | **0.23** | **1.12** | **9.37** | **169** | **45.40** | **48.50** | **18.07** | **372** | **17.93** | **14.83** | **956** | **5.53** |
| SD | | | **1.27** | **2.43** | **2.10** | **0.51** | **0.72** | **0.12** | **0.45** | **0.32** | **4.62** | **0.87** | **0.70** | **0.59** | **8.89** | **0.21** | **0.15** | **150.77** | **0.15** |
| SE | | | **0.73** | **1.40** | **1.21** | **0.30** | **0.41** | **0.07** | **0.26** | **0.18** | **2.67** | **0.50** | **0.40** | **0.34** | **5.13** | **0.12** | **0.09** | **87.05** | **0.09** |
| 4 | 4 | Up284, 40 mg/kg IP | 4.20 | 55.5 | 6.8 | 37.7 | 2.33 | 0.29 | 1.58 | 8.72 | 156 | 42.40 | 48.60 | 17.80 | 367 | 18.20 | 15.00 | 960 | 5.40 |
| 18 | 18 |  | 2.20 | 71.1 | 3.3 | 25.6 | 1.56 | 0.07 | 0.56 | 9.54 | 168 | 44.80 | 46.90 | 17.60 | 375 | 17.10 | 14.50 | 1154 | 5.30 |
| 22 | 22 |  | 4.60 | 53.4 | 6.2 | 40.4 | 2.46 | 0.29 | 1.86 | 9.32 | 164 | 44.40 | 47.70 | 17.60 | 368 | 17.50 | 14.70 | 890 | 5.50 |
| Mean | | | **3.67** | **60.00** | **5.43** | **34.57** | **2.12** | **0.21** | **1.34** | **9.19** | **163** | **43.87** | **47.73** | **17.67** | **370** | **17.60** | **14.73** | **1001** | **5.40** |
| SD | | | **1.29** | **9.67** | **1.87** | **7.88** | **0.48** | **0.12** | **0.68** | **0.42** | **6.11** | **1.29** | **0.85** | **0.12** | **4.36** | **0.56** | **0.25** | **136.77** | **0.10** |
| SE | | | **0.74** | **5.58** | **1.08** | **4.55** | **0.28** | **0.07** | **0.39** | **0.25** | **3.53** | **0.74** | **0.49** | **0.07** | **2.52** | **0.32** | **0.15** | **78.96** | **0.06** |
| **P (t-test, compared to Vehicle)** | | | 0.7654 | 0.9739 | 0.4897 | 0.8318 | 0.8023 | 0.9019 | 0.6742 | 0.6015 | 0.2464 | 0.1625 | 0.2944 | 0.3105 | 0.7440 | 0.3864 | 0.5879 | 0.7193 | 0.2746 |

Final blood parameters values PO

| Cage # | mouse # | Compound, dose | WBC, 10^9^/L | LYM, % | MID, % | GRAN, % | LYM, 109/L | MID, 109/L | GRAN, 109/L | RBC, 10^12^/L | HGB, g/L | HCT, % | MCV, fL | MCH, pg | MCHC, g/L | RDW-SD, fL | RDW-CV, % | PLT, 10^9^/L | MPV, fL |
| --- | --- | --- | --- | --- | --- | --- | --- | --- | --- | --- | --- | --- | --- | --- | --- | --- | --- | --- | --- |
| 10 | 10 | Vehicle PO | 1.40 | 72.0 | 5.7 | 22.3 | 1.01 | 0.08 | 0.31 | 9.10 | 168 | 44.40 | 48.80 | 18.40 | 377 | 17.90 | 14.70 | 840 | 5.40 |
| 20 | 20 |  | 3.80 | 57.1 | 8.6 | 34.3 | 2.17 | 0.33 | 1.30 | 9.90 | 184 | 48.00 | 48.40 | 18.70 | 386 | 18.20 | 15.10 | 864 | 5.50 |
| 30 | 30 |  | 4.80 | 54.0 | 12.1 | 33.9 | 2.59 | 0.58 | 1.63 | 9.12 | 168 | 47.00 | 51.60 | 18.30 | 356 | 19.40 | 15.00 | 890 | 5.50 |
| Mean | | | **3.33** | **61.03** | **8.80** | **30.17** | **1.92** | **0.33** | **1.08** | **9.37** | **173** | **46.47** | **49.60** | **18.47** | **373** | **18.50** | **14.93** | **865** | **5.47** |
| SD | | | **1.75** | **9.62** | **3.20** | **6.82** | **0.82** | **0.25** | **0.69** | **0.46** | **9.24** | **1.86** | **1.74** | **0.21** | **15.39** | **0.79** | **0.21** | **25.01** | **0.06** |
| SE | | | **1.01** | **5.56** | **1.85** | **3.94** | **0.47** | **0.14** | **0.40** | **0.26** | **5.33** | **1.07** | **1.01** | **0.12** | **8.89** | **0.46** | **0.12** | **14.44** | **0.03** |
| 5 | 5 | Up284, 200 mg/kg PO | 3.40 | 68.5 | 8.6 | 22.9 | 2.33 | 0.29 | 0.78 | 9.22 | 166 | 46.60 | 50.60 | 17.90 | 354 | 19.20 | 15.10 | 888 | 5.30 |
| 34 | 34 |  | 3.60 | 66.7 | 8.1 | 25.2 | 2.40 | 0.29 | 0.91 | 8.84 | 166 | 45.00 | 50.90 | 18.90 | 371 | 18.90 | 14.90 | 822 | 5.40 |
| 37 | 37 |  | 3.80 | 71.8 | 8.2 | 20.0 | 2.73 | 0.31 | 0.76 | 8.96 | 164 | 45.20 | 50.50 | 18.20 | 360 | 18.60 | 14.80 | 1116 | 5.50 |
| Mean | | | **3.60** | **69.00** | **8.30** | **22.70** | **2.49** | **0.30** | **0.82** | **9.01** | **165** | **45.60** | **50.67** | **18.33** | **362** | **18.90** | **14.93** | **942** | **5.40** |
| SD | | | **0.20** | **2.59** | **0.26** | **2.61** | **0.21** | **0.01** | **0.08** | **0.19** | **1.15** | **0.87** | **0.21** | **0.51** | **8.62** | **0.30** | **0.15** | **154.26** | **0.10** |
| SE | | | **0.12** | **1.49** | **0.15** | **1.50** | **0.12** | **0.01** | **0.05** | **0.11** | **0.67** | **0.50** | **0.12** | **0.30** | **4.98** | **0.17** | **0.09** | **89.06** | **0.06** |
| **P (t-test, compared to Vehicle)** | | | 0.8058 | 0.2384 | 0.8010 | 0.1510 | 0.3140 | 0.8429 | 0.5412 | 0.2695 | 0.2109 | 0.5051 | 0.3521 | 0.6981 | 0.3283 | 0.4601 | 1.0000 | 0.4397 | 0.3739 |
