# Supplementary material for: Development and anticancer properties of Up284, a spirocyclic candidate ADRM1/RPN13 inhibitor
Source: PLoS One. 2023 Jun 14;18(6):e0285221. doi: 10.1371/journal.pone.0285221 (PMC10266688; doi:10.1371/journal.pone.0285221)
Supplement: S8 Table — (DOCX) [file pone.0285221.s011.docx]

Table S8. Repeat dose toxicity study for Up284 and bortezomib (IV in 5 mL/kg) in female CD1 mice (9 weeks old).

| Day # | Group 1 | Group 2 | Group 3 |
| --- | --- | --- | --- |
|  | Vehicle (25% 2-HPβCD in water) | Up284 | Bortezomib in saline |
|  | Dose: | 20 mg/kg | 1 mg/kg |
|  | Number of mice: 3 | 3 | 3 |
| 0 | X Gc W | X Gc W | X Gc W |
| 1 | Gc W | Gc W | Gc W |
| 2 | Gc W | Gc W | Gc W |
| 3 | X Gc W | X Gc W | X Gc W |
| 4 | Gc W | Gc W | Gc W |
| 5 | Gc W | Gc W | Gc W |
| 6 | X Gc W | X Gc W | X Gc W |
| 7 | Bt Gc W | Bt Gc W | Bt Gc W |

Bt - terminal bleeding of survived mice for hematology and clinical chemistry (ALAT, ASAT, ALP, LDH, CK, Creatinine, Urea, GGT, and TP)

Gc - general condition observation

W – weighing

X - treatment with compound or vehicle
