# Supplementary material for: Development and anticancer properties of Up284, a spirocyclic candidate ADRM1/RPN13 inhibitor
Source: PLoS One. 2023 Jun 14;18(6):e0285221. doi: 10.1371/journal.pone.0285221 (PMC10266688; doi:10.1371/journal.pone.0285221)
Supplement: S9 Table — (DOCX) [file pone.0285221.s012.docx]

Table S9. Body weights in repeat dose toxicity study for Up284 and bortezomib in female CD1 mice (9 weeks old).

| Cage # | Mouse # | Compound, dose | Body weight at different days of the study, g | | | | | | | | Body weight change*, % |
| --- | --- | --- | --- | --- | --- | --- | --- | --- | --- | --- | --- |
|  |  |  | 0 | 1 | 2 | 3 | 4 | 5 | 6 | 7 |  |
| 3 | 3 | Vehicle | 26.8 | 26.3 | 26.5 | 27.0 | 26.6 | 26.4 | 26.7 | 27.2 | 1.49 |
| 10 | 10 |  | 24.7 | 25.1 | 25.8 | 25.0 | 25.5 | 26.0 | 25.8 | 25.3 | 2.43 |
| 11 | 11 |  | 26.8 | 27.0 | 28.2 | 28.1 | 27.9 | 27.5 | 27.1 | 27.8 | 3.73 |
| Mean | | | **26.10** | **26.13** | **26.83** | **26.70** | **26.67** | **26.63** | **26.53** | **26.77** | **2.55** |
| SD | | | **1.21** | **0.96** | **1.23** | **1.57** | **1.20** | **0.78** | **0.67** | **1.31** | **1.12** |
| SE | | | **0.70** | **0.55** | **0.71** | **0.91** | **0.69** | **0.45** | **0.38** | **0.75** | **0.65** |
| 5 | 5 | Up284, 20 mg/kg | 26.1 | 27.5 | 27.9 | 26.4 | 27.0 | 26.8 | 27.6 | 26.8 | 2.68 |
| 6 | 6 |  | 25.6 | 26.2 | 26.3 | 25.4 | 26.0 | 26.2 | 26.1 | 26.7 | 4.30 |
| 9 | 9 |  | 28.0 | 27.8 | 28.0 | 27.8 | 28.0 | 27.8 | 28.5 | 28.0 | 0.00 |
| Mean | | | **26.57** | **27.17** | **27.40** | **26.53** | **27.00** | **26.93** | **27.40** | **27.17** | **2.33** |
| SD | | | **1.27** | **0.85** | **0.95** | **1.21** | **1.00** | **0.81** | **1.21** | **0.72** | **2.17** |
| SE | | | **0.73** | **0.49** | **0.55** | **0.70** | **0.58** | **0.47** | **0.70** | **0.42** | **1.25** |
| **P (t-test, compared to Vehicle)** | | | 0.6687 | 0.2356 | 0.5634 | 0.8912 | 0.7306 | 0.6671 | 0.3389 | 0.6666 | 0.8812 |
| **P (t-test, compared to bortezomib)** | | | 0.8865 | 0.5577 | 0.9187 | 0.8642 | 0.6739 | 0.9419 | 0.7930 | 0.8385 | 0.8635 |
| 1 | 1 | Bortezomib, 1 mg/kg | 27.4 | 28.1 | 28.7 | 27.5 | 28.0 | 28.4 | 28.7 | 28.5 | 4.01 |
| 4 | 4 |  | 25.9 | 25.4 | 26.2 | 25.5 | 25.4 | 26.0 | 26.1 | 25.7 | -0.77 |
| 7 | 7 |  | 26.0 | 26.2 | 27.0 | 26.1 | 26.3 | 26.6 | 26.5 | 26.7 | 2.69 |
| Mean | | | **26.43** | **26.57** | **27.30** | **26.37** | **26.57** | **27.00** | **27.10** | **26.97** | **1.98** |
| SD | | | **0.84** | **1.39** | **1.28** | **1.03** | **1.32** | **1.25** | **1.40** | **1.42** | **2.47** |
| SE | | | **0.48** | **0.80** | **0.74** | **0.59** | **0.76** | **0.72** | **0.81** | **0.82** | **1.43** |
| **P (t-test, compared to Vehicle)** | | | 0.7153 | 0.6794 | 0.6726 | 0.7737 | 0.9274 | 0.6882 | 0.5610 | 0.8661 | 0.7334 |

* Body weight change compared to the initial weight was calculated for the 7th day of the study
