# Supplementary material for: Development and anticancer properties of Up284, a spirocyclic candidate ADRM1/RPN13 inhibitor
Source: PLoS One. 2023 Jun 14;18(6):e0285221. doi: 10.1371/journal.pone.0285221 (PMC10266688; doi:10.1371/journal.pone.0285221)
Supplement: S10 Table — (DOCX) [file pone.0285221.s013.docx]

Table S10. Clinical observations in repeat dose toxicity study for Up284 and bortezomib in female CD1 mice (9 weeks old).

| Cage # | Mouse # | Compound, dose | Days and hours of observations | | | | | | | | | | | |
| --- | --- | --- | --- | --- | --- | --- | --- | --- | --- | --- | --- | --- | --- | --- |
|  |  |  | 0 | | | | | 1 | 2 | 3 | 4 | 5 | 6 | 7 |
|  |  |  | 0 | 0.5 h | 2 h | 4 h | 6 h |  |  |  |  |  |  |  |
| 3 | 3 | Vehicle | 0 | 0 | 0 | 0 | 0 | 0 | 0 | 0 | 0 | 0 | 0 | 0 |
| 10 | 10 |  | 0 | 0 | 0 | 0 | 0 | 0 | 0 | 0 | 0 | 0 | 0 | 0 |
| 11 | 11 |  | 0 | 0 | 0 | 0 | 0 | 0 | 0 | 0 | 0 | 0 | 0 | 0 |
| 5 | 5 | Up284, 20 mg/kg | 0 | 0 | 0 | 0 | 0 | 0 | 0 | 0 | 0 | 0 | 0 | 0 |
| 6 | 6 |  | 0 | 0 | 0 | 0 | 0 | 0 | 0 | 0 | 0 | 0 | 0 | 0 |
| 9 | 9 |  | 0 | 0 | 0 | 0 | 0 | 0 | 0 | 0 | 0 | 0 | 0 | 0 |
| 1 | 1 | Bortezomib, 1 mg/kg | 0 | 0 | 0 | 0 | 0 | 0 | 0 | 0 | 0 | 0 | 0 | 0 |
| 4 | 4 |  | 0 | 0 | 0 | 0 | 0 | 0 | 0 | 0 | 0 | 0 | 0 | 0 |
| 7 | 7 |  | 0 | 0 | 0 | 0 | 0 | 0 | 0 | 0 | 0 | 0 | 0 | 0 |

Key: 0 – good appearance
