# Supplementary material for: Development and anticancer properties of Up284, a spirocyclic candidate ADRM1/RPN13 inhibitor
Source: PLoS One. 2023 Jun 14;18(6):e0285221. doi: 10.1371/journal.pone.0285221 (PMC10266688; doi:10.1371/journal.pone.0285221)
Supplement: S11 Table — (DOCX) [file pone.0285221.s014.docx]

Table S11. Hematologic parameters in repeat dose toxicity study for Up284 and bortezomib in female CD1 mice (9 weeks old).

| Cage # | mouse # | Compound, dose | WBC, 10^9^/L | LYM, % | MID, % | GRAN, % | LYM, 109/L | MID, 109/L | GRAN, 109/L | RBC, 10^12^/L | HGB, g/L | HCT, % | MCV, fL | MCH, pg | MCHC, g/L | RDW-SD, fL | RDW-CV, % | PLT, 10^9^/L | MPV, fL |
| --- | --- | --- | --- | --- | --- | --- | --- | --- | --- | --- | --- | --- | --- | --- | --- | --- | --- | --- | --- |
| 3 | 3 | Vehicle | 3.40 | 66.9 | 9.2 | 23.9 | 2.27 | 0.31 | 0.81 | 9.68 | 168 | 46.60 | 48.10 | 17.40 | 361 | 17.40 | 14.40 | 1070 | 5.40 |
| 10 | 10 |  | 2.20 | 60.3 | 8.0 | 31.7 | 1.33 | 0.18 | 0.70 | 9.86 | 180 | 49.40 | 50.10 | 18.20 | 363 | 18.20 | 14.50 | 1092 | 5.60 |
| 11 | 11 |  | 2.60 | 59.7 | 8.2 | 32.1 | 1.55 | 0.21 | 0.83 | 9.76 | 176 | 47.60 | 48.80 | 18.10 | 371 | 17.80 | 14.60 | 888 | 5.70 |
| Mean | | | **2.73** | **62.30** | **8.47** | **29.23** | **1.72** | **0.23** | **0.78** | **9.77** | **175** | **47.87** | **49.00** | **17.90** | **365** | **17.80** | **14.50** | **1017** | **5.57** |
| SD | | | **0.61** | **3.99** | **0.64** | **4.62** | **0.50** | **0.07** | **0.07** | **0.09** | **6.11** | **1.42** | **1.01** | **0.44** | **5.29** | **0.40** | **0.10** | **111.97** | **0.15** |
| SE | | | **0.35** | **2.31** | **0.37** | **2.67** | **0.29** | **0.04** | **0.04** | **0.05** | **3.53** | **0.82** | **0.59** | **0.25** | **3.06** | **0.23** | **0.06** | **64.65** | **0.09** |
| 5 | 5 | Up284, 20 mg/kg | 2.20 | 57.3 | 7.2 | 35.5 | 1.26 | 0.16 | 0.78 | 8.70 | 154 | 42.20 | 48.60 | 17.80 | 366 | 17.00 | 14.00 | 1102 | 5.40 |
| 6 | 6 |  | 3.80 | 64.9 | 7.3 | 27.8 | 2.47 | 0.28 | 1.06 | 9.22 | 164 | 44.20 | 48.00 | 17.80 | 371 | 17.40 | 14.50 | 902 | 5.40 |
| 9 | 9 |  | 4.80 | 69.3 | 7.7 | 23.0 | 3.33 | 0.37 | 1.10 | 8.92 | 160 | 45.40 | 51.00 | 18.00 | 352 | 18.20 | 14.20 | 1412 | 5.40 |
| Mean | | | **3.60** | **63.83** | **7.40** | **28.77** | **2.35** | **0.27** | **0.98** | **8.95** | **159** | **43.93** | **49.20** | **17.87** | **363** | **17.53** | **14.23** | **1139** | **5.40** |
| SD | | | **1.31** | **6.07** | **0.26** | **6.31** | **1.04** | **0.11** | **0.17** | **0.26** | **5.03** | **1.62** | **1.59** | **0.12** | **9.85** | **0.61** | **0.25** | **256.97** | **0.00** |
| SE | | | **0.76** | **3.50** | **0.15** | **3.64** | **0.60** | **0.06** | **0.10** | **0.15** | **2.91** | **0.93** | **0.92** | **0.07** | **5.69** | **0.35** | **0.15** | **148.36** | **0.00** |
| **P (t-test, compared to Vehicle)** | | | 0.3581 | 0.7333 | 0.0565 | 0.9226 | 0.3941 | 0.6636 | 0.1429 | **0.0068** | **0.0284** | **0.0339** | 0.8631 | 0.9043 | 0.7722 | 0.5614 | 0.1633 | 0.4929 | 0.1318 |
| **P (t-test, compared to bortezomib)** | | | 0.9495 | 0.0724 | 0.5876 | 0.0678 | 0.7377 | 0.5472 | 0.0641 | 0.5089 | 0.7090 | 0.5616 | 0.8708 | **0.0020** | 0.0801 | 0.6026 | 0.2417 | 0.1089 | **0.0065** |
| 1 | 1 | Bortezomib, 1 mg/kg | 2.80 | 69.8 | 9.0 | 21.2 | 1.95 | 0.25 | 0.59 | 9.16 | 168 | 45.00 | 49.10 | 18.40 | 375 | 17.80 | 14.50 | 840 | 5.70 |
| 4 | 4 |  | 3.00 | 74.5 | 6.0 | 19.5 | 2.24 | 0.18 | 0.59 | 8.36 | 154 | 40.60 | 48.50 | 18.50 | 382 | 17.70 | 14.60 | 822 | 5.60 |
| 7 | 7 |  | 4.80 | 78.0 | 5.1 | 16.9 | 3.74 | 0.24 | 0.81 | 8.72 | 162 | 43.20 | 49.50 | 18.60 | 375 | 17.70 | 14.30 | 838 | 5.80 |
| Mean | | | **3.53** | **74.10** | **6.70** | **19.20** | **2.64** | **0.23** | **0.66** | **8.75** | **161** | **42.93** | **49.03** | **18.50** | **377** | **17.73** | **14.47** | **833** | **5.70** |
| SD | | | **1.10** | **4.11** | **2.04** | **2.17** | **0.96** | **0.04** | **0.13** | **0.40** | **7.02** | **2.21** | **0.50** | **0.10** | **4.04** | **0.06** | **0.15** | **9.87** | **0.10** |
| SE | | | **0.64** | **2.38** | **1.18** | **1.25** | **0.56** | **0.02** | **0.07** | **0.23** | **4.06** | **1.28** | **0.29** | **0.06** | **2.33** | **0.03** | **0.09** | **5.70** | **0.06** |
| **P (t-test, compared to Vehicle)** | | | 0.3331 | **0.0235** | 0.2261 | **0.0272** | 0.2123 | 0.8663 | 0.2382 | **0.0126** | 0.0682 | **0.0313** | 0.9618 | 0.0808 | **0.0326** | 0.7893 | 0.7676 | **0.0476** | 0.2746 |
