# Supplementary material for: Development and anticancer properties of Up284, a spirocyclic candidate ADRM1/RPN13 inhibitor
Source: PLoS One. 2023 Jun 14;18(6):e0285221. doi: 10.1371/journal.pone.0285221 (PMC10266688; doi:10.1371/journal.pone.0285221)
Supplement: S12 Table — (DOCX) [file pone.0285221.s015.docx]

Table S12. Blood chemistry parameters in repeat dose toxicity study for Up284 and bortezomib in female CD1 mice (9 weeks old).

| Cage # | Mouse # | Compound, dose | ALAT, U/l | ASAT, U/l | ALP, U/l | LDH, U/l | GGT, U/l | CK, U/l | Creatinine, µmol/l | Urea, mmol/l | TP,g/l |
| --- | --- | --- | --- | --- | --- | --- | --- | --- | --- | --- | --- |
| 3 | 3 | Vehicle | 29.46 | 110.4 | 120 | 1100 | 4.45 | 960 | 25.4 | 5.48 | 50.6 |
| 10 | 10 |  | 30.84 | 89.1 | 119 | 816 | 5.18 | 408 | 24.9 | 6.97 | 58.9 |
| 11 | 11 |  | 28.43 | 107.6 | 102 | 849 | 5.10 | 676 | 25.1 | 6.44 | 52.2 |
| Mean | | | **29.58** | **102.38** | **113.82** | **958.25** | **4.82** | **684.14** | **25.16** | **6.30** | **53.90** |
| SD | | | **1.21** | **11.59** | **9.88** | **201.14** | **0.51** | **390.39** | **0.39** | **0.76** | **4.41** |
| SE | | | **0.70** | **6.69** | **5.71** | **142.23** | **0.36** | **276.05** | **0.27** | **0.44** | **2.55** |
| 5 | 5 | Up284, 20 mg/kg | 24.69 | 81.3 | 93 | 1063 | 5.29 | 773 | 26.0 | 6.50 | 50.4 |
| 6 | 6 |  | 23.85 | 99.8 | 93 | 692 | 5.37 | 505 | 24.6 | 7.88 | 53.1 |
| 9 | 9 |  | 32.08 | 96.1 | 97 | 946 | 5.25 | 653 | 27.0 | 7.86 | 53.5 |
| Mean | | | **26.87** | **92.38** | **94.53** | **877.58** | **5.33** | **638.73** | **25.34** | **7.41** | **52.33** |
| SD | | | **4.53** | **9.79** | **2.57** | **262.52** | **0.05** | **189.55** | **0.99** | **0.79** | **1.66** |
| SE | | | **2.61** | **5.65** | **1.49** | **185.63** | **0.04** | **134.03** | **0.70** | **0.45** | **0.96** |
| **P (t-test, compared to Vehicle)** | | | 0.3737 | 0.3171 | **0.0308** | 0.8857 | 0.1679 | 0.8421 | 0.3521 | 0.1512 | 0.5937 |
| **P (t-test, compared to bortezomib)** | | | 0.5595 | 0.5716 | 0.4370 | 0.3334 | 0.2913 | 0.8773 | 0.1572 | 0.0893 | 0.0805 |
| 1 | 1 | Bortezomib, 1 mg/kg | 16.23 | 65.7 | 79 | 615 | 5.29 | 174 | 23.4 | 4.87 | 51.2 |
| 4 | 4 |  | 23.80 | 86.9 | 84 | 941 | 4.94 | 409 | 23.9 | 6.73 | 46.0 |
| 7 | 7 |  | 31.03 | 102.4 | 102 | 647 | 5.25 | 1191 | 25.5 | 3.27 | 46.0 |
| Mean | | | **23.69** | **84.97** | **88.27** | **734.41** | **5.16** | **591.48** | **24.27** | **4.96** | **47.72** |
| SD | | | **7.40** | **18.41** | **12.30** | **179.76** | **0.19** | **532.58** | **1.11** | **1.73** | **3.00** |
| SE | | | **4.28** | **10.63** | **7.10** | **103.78** | **0.11** | **307.48** | **0.64** | **1.00** | **1.73** |
| **P (t-test, compared to Vehicle)** | | | 0.2455 | 0.2379 | **0.0486** | 0.2434 | 0.3798 | 0.8081 | 0.2490 | 0.2873 | 0.1151 |
