# Supplementary material for: Development and anticancer properties of Up284, a spirocyclic candidate ADRM1/RPN13 inhibitor
Source: PLoS One. 2023 Jun 14;18(6):e0285221. doi: 10.1371/journal.pone.0285221 (PMC10266688; doi:10.1371/journal.pone.0285221)
Supplement: S13 Table — (DOCX) [file pone.0285221.s016.docx]

Table S13. Study to determine the pharmacokinetic characteristics of compound Up284 in male CD1 mice (8 weeks old) following intravenous (IV), intraperitoneal (IP) and per oral (PO) administration.

| Number of Animals | Compound ID | Formulation | Delivery Route | Target Dose Level (mg/kg) | Target Dose Concentration (mg/ml) | Target Dose Volume (ml/kg) |
| --- | --- | --- | --- | --- | --- | --- |
| 36 | Up284 | DMSO (50 uL)+25% b-  hydroxypropyl cyclodextrin in water | IV | 5 | 1 | 5 |
| 1 | Vehicle dosed |  | IV | 0 | 0 | 5 |
| 36 | Up284 | DMSO (50 uL)+25% b-  hydroxypropyl cyclodextrin in water | IP | 20 | 4 | 5 |
| 1 | Vehicle dosed |  | IP | 0 | 0 | 5 |
| 32 | Up284 | DMSO (50 uL)+25% b-  hydroxypropyl cyclodextrin in water | PO | 50 | 10 | 5 |
| 1 | Vehicle dosed |  | PO | 0 | 0 | 5 |
