# Supplementary material for: Development and anticancer properties of Up284, a spirocyclic candidate ADRM1/RPN13 inhibitor
Source: PLoS One. 2023 Jun 14;18(6):e0285221. doi: 10.1371/journal.pone.0285221 (PMC10266688; doi:10.1371/journal.pone.0285221)
Supplement: S14 Table — (DOCX) [file pone.0285221.s017.docx]

Table S14. Plasma concentrations of Up284 in male CD1 mice following IV (5 mg/kg) administration.

|  | | | | | | | |
| --- | --- | --- | --- | --- | --- | --- | --- |
| **Sample collection time point,**  **min** | **Plasma concentration (ng/ml)** | | | | | | |
|  | **Group A** | **Group B** | **Group C** | **Group D** | **Mean** | **SD** | **SE** |
| 0 | BQL |  |  |  | **BQL** | ND | ND |
| 5 | 1615 | 1306 | 1278 | 1605 | **1451** | 184 | 92 |
| 15 | 1502 | 1593 | 1472 | 1463 | **1508** | 59 | 30 |
| 30 | 1526 | 1037 | 1261 | 1199 | **1256** | 203 | 102 |
| 60 | 793 | 812 | 794 | 636* | **800** | 11 | 6 |
| 120 | 670 | 671 | 527* | 665 | **669** | 3 | 2 |
| 240 | 504 | 365 | 477 | 338 | **421** | 82 | 41 |
| 360 | 256 | 217 | 228 | 249 | **238** | 18 | 9 |
| 480 | 250 | 193 | 199 | 117 | **190** | 55 | 27 |
| 1440 | BQL | 141 | 100 | 223 | **116** | 93 | 46 |

BQL - Below the lower limit of quantitation (LLOQ)

ND - Not determined

*Grubbs’ outlier test: Significant outlier. P < 0.05
