# Supplementary material for: Development and anticancer properties of Up284, a spirocyclic candidate ADRM1/RPN13 inhibitor
Source: PLoS One. 2023 Jun 14;18(6):e0285221. doi: 10.1371/journal.pone.0285221 (PMC10266688; doi:10.1371/journal.pone.0285221)
Supplement: S15 Table — (DOCX) [file pone.0285221.s018.docx]

| **Sample collection**  **time point, min** | **Plasma concentration (ng/ml)** | | | | | | |
| --- | --- | --- | --- | --- | --- | --- | --- |
|  | **Group A** | **Group B** | **Group C** | **Group D** | **Mean** | **SD** | **SE** |
| 0 | BQL |  |  |  | **BQL** | ND | ND |
| 5 | 503 | 865 | 1166 | 1063 | **899** | 292 | 146 |
| 15 | 2475 | 1865 | 2398 | 1985 | **2181** | 301 | 150 |
| 30 | 1344 | 3322 | 2468 | 2724 | **2465** | 828 | 414 |
| 60 | 2399 | 2442 | 2380 | 2367 | **2397** | 33 | 16 |
| 120 | 1951 | 2366 | 2232 | 1997 | **2137** | 196 | 98 |
| 240 | 1536* | 1756 | 1722 | 1749 | **1742** | 18 | 10 |
| 360 | 240 | 1124 | 495 | 1296 | **789** | 502 | 251 |
| 480 | 1103 | 758 | 770 | 292 | **731** | 333 | 167 |
| 1440 | 750 | 416 | 713 | 561 | **610** | 153 | 77 |

Table S15. Plasma concentrations for Up284 in CD-1 mice following IP (20 mg/kg) administration.
