# Supplementary material for: Development and anticancer properties of Up284, a spirocyclic candidate ADRM1/RPN13 inhibitor
Source: PLoS One. 2023 Jun 14;18(6):e0285221. doi: 10.1371/journal.pone.0285221 (PMC10266688; doi:10.1371/journal.pone.0285221)
Supplement: S16 Table — (DOCX) [file pone.0285221.s019.docx]

Table S16. Plasma concentrations of Up284 in CD-1 mice following PO (50 mg/kg) administration.

| **Sample collection**  **time point, min** | **Plasma concentration (ng/ml)** | | | | | | |
| --- | --- | --- | --- | --- | --- | --- | --- |
|  | **Group A** | **Group B** | **Group C** | **Group D** | **Mean** | **SD** | **SE** |
| 0 | BQL |  |  |  | **BQL** | ND | ND |
| 15 | 1200 | 1259 | 2379 | 2486 | **1831** | 696 | 348 |
| 30 | 1462 | 1112 | 995 | 1560 | **1282** | 271 | 136 |
| 60 | 1565 | 1676 | 1298 | 1181 | **1430** | 230 | 115 |
| 120 | 1649* | 1235 | 1195 | 1216 | **1215** | 20 | 12 |
| 240 | 1399 | 366 | 838 | 587 | **798** | 445 | 222 |
| 360 | 318 | 539 | 550 | 486 | **473** | 107 | 54 |
| 480 | 941 | 597 | 376 | 440 | **589** | 253 | 126 |
| 1440 | 189 | 846 | 317 | 347 | **425** | 289 | 145 |

BQL - Below the lower limit of quantitation (LLOQ)

ND - Not determined

*Grubbs’ outlier test: Significant outlier. P < 0.05
