# Supplementary material for: Development and anticancer properties of Up284, a spirocyclic candidate ADRM1/RPN13 inhibitor
Source: PLoS One. 2023 Jun 14;18(6):e0285221. doi: 10.1371/journal.pone.0285221 (PMC10266688; doi:10.1371/journal.pone.0285221)
Supplement: S17 Table — (DOCX) [file pone.0285221.s020.docx]

Table S17. Comparison of selected pharmacokinetic parameters for Up284 in male CD1 mice.

| Sample | Administration | Dose, mg/kg | Pharmacokinetic Parameters | | | | | | | |
| --- | --- | --- | --- | --- | --- | --- | --- | --- | --- | --- |
|  |  |  | Tmax, min | Cmax, ng/ml | AUC0→t min (AUClast) ng*min/ml | AUC0→∞ (AUCINF_obs) ng*min/ml | T1/2  (HL_Lambda_z), min | Kel (Lambda_z), min^-1^ | Vd (Vz_obs) ml/kg | bioavailability,  % |
| Plasma | IV | 5 | - | 1510 | 395000 | 586000 | 1140 | 0.000606 | 10000 | 100 |
|  | PO | 50 | 15.0 | 1830 | 904000 | 1370000 | 764 | 0.000907 | ND | 23 |
|  | IP | 20 | 30.0 | 2460 | 1380000 | 4170000 | 3170 | 0.000219 | ND | 78 |
