# Supplementary material for: Development and anticancer properties of Up284, a spirocyclic candidate ADRM1/RPN13 inhibitor
Source: PLoS One. 2023 Jun 14;18(6):e0285221. doi: 10.1371/journal.pone.0285221 (PMC10266688; doi:10.1371/journal.pone.0285221)
Supplement: S1 File — Description of chemical synthesis of compounds in Table 1. (DOCX) [file pone.0285221.s021.docx]

**Supplemental information**

**Description of Synthesis**

The following abbreviations are used and have the indicated definitions: MHz is megahertz (frequency), m is multiplet, d is doublet, s is singlet, CDCl3 is deuterated chloroform, DMSO-d6 is deuterated DMSO, min is minutes, h is hours, g is grams, mg is milli grams, mmol is milli moles, mL is milliliters, µL is micro liters,

N is normality, M is molarity, µM is micro molar, nM is nano molar, ^0^C is centigrade, TLC is thin layer chromatography, NMR is Nuclear Magnetic Resonance, ESI MS is Electro spray ionization mass spectrometry, DMSO is dimethyl sulfoxide, DCM is dichloromethane, DMF is N,N-dimethyl formamide, THF is tetrahydrofuran, NaHCO3 is sodium bicarbonate, RT is room temperature, EtOH is ethanol, NaOH is sodium hydroxide, KOH is potassium hydroxide, Et3N is triethyl amine, DIPEA is diisopropylethylamine, HCl is hydrogen chloride or hydrochloric acid, AcOH is acetic acid, TFA is trifluoro aceticacid, HOBT is N-hydroxy-benzotriazole, HBTU is 2-(1H-benzotriazol-1-yl)-1,1,3,3-tetramethyluronium hexafluorophosphate, Boc-Phe-OH is  ***N*-(*tert*-Butoxycarbonyl)-L-phenylalanine, Boc-L-phenylalanine,**

**General Synthetic Procedure A:**

To a stirred solution of compound GA (1 eq) in any alcohol (example: Ethanol) were added a base such as NaOH (4 eq) or KOH (4 eq) dissolved in water and an aldehyde (RCHO) (2eq) and the reaction mixture was stirred at room temperature until TLC shows completion of the reaction. Water was added to the reaction mixture and the precipitated yellow solid was filtered, washed with water and dried under vacuum and purified by column chromatography over silica gel using ethyl acetate: hexanes mixture or crystallized in organic solvents to give the title compound GB which was dissolved in dioxane or any other solvents and treated with an acid (example 4M HCl in dioxane) and stirred at room temperature until the TLC show the completion of the reaction. Solvents were removed under vacuum and the solids were precipitated with diethyl ether or hexanes or any other solvents and dried under vacuum to give the title compound GC.

**General Synthetic Procedure B:**

Compound GD (1 eq) in THF or DCM (dichloromethane) was treated with Boron trifluoride diethyl etherate (4-8 eq) dropwise at 0^0^C, then aldehyde (2eq) was added to the reaction mixture in one portion and stirred overnight at room temperature. The reaction was quenched with a 10% aq. sodium bicarbonate solution and the yellow solid precipitate was filtered, washed with water and ethanol afforded compound GC. The typical yield in this procedure is 30-55%.

**General synthetic procedure C:**

Compound GD (0.5 g, 1 eq) was dissolved in 10 mL acetic acid and added RCHO (2 eq). To this reaction mixture either dryHCL (generated from NaCl and H2SO4) gas bubbled for 15 min or direct H_2_SO_4_ (2 mL) was added in two portions and the reaction mixture stirred for overnight at room temperature. Solids were filtered off and washed with cold ethanol and dried under vacuum to afford compound GC. Typical yield in this procedure is 45-66%.

**Examples:**

Synthesis of Up284: To a stirred solution of 2-Oxo-7-azaspiro[3.5]nonane-7-carboxylate tert-butyl ester **1** (1.008, 1 eq) in 20 mL Ethanol were added aq.30% Sodium Hydroxide solution (4 eq, 2.4 mL) and 4-cyano benzaldehyde (1.18 g, 2 eq) at room temperature and stirring continued for 120 min. Top the reaction mixture water was added and the precipitated yellow solid was filtered, dried under vacuum and purified by column chromatography over silica gel using hexanes: ethyl acetate (8:2) as eluents. Removal of solvents under reduced pressure afforded Up283 as yellow solid (1.6 g, 82.4%). To a stirred solution of Up283 (1.2 g) in dioxane (10 mL) was added 4M HCl in dioxane (10 mL) and stirring continued for 60 min at room temperature. Solvents were removed under vacuum and the resultant yellow precipitate was precipitated with diethyl ether and dried under vacuum to afford **Up284** (0.78 g, 75.7%) as yellow powder. ^1^HNMR (DMSO-d6, 400 MHz): δ 9.09 (s, 1H); 8.3 (d, 4H); 7.93 (d, 4H); 7.16 (s,2H); 3.46 (s, 4H); 2.2 (s, 4H). ESI MS (m/z): 366 (M+1)

Synthesis of Up285: To a stirred solution of Up284 (0.2 g, 1 eq) in dichloromethane (10 mL) was added diisopropyl ethylamine (0.27 mL, 3 eq) followed by acryloyl chloride (43 µL, 1.05eq) at 0^0^C and stirred for 60 min at room temperature. Reaction completion was monitored by TLC. The reaction mixture was washed with saturated sodium bicarbonate aqueous solution (20mL), extracted the compound with dichloromethane, dried over sodium sulfate, filtered and concentrated. The crude product was purified by column chromatography over silica gel using hexanes:ethyl acetate (4:6) as eluents to give the title compound **Up285** (76%, 0.154 g) as an yellow powder. ^1^HNMR (CDCl_3_, 400 MHz): 8.1 (d, 4H); 7.72 (d, 4H); 6.67-6.72 (m, 3H); 6.23-6.45 (m, 1H); 5.71-5.83 (m, 1H); 3.66-3.94 (m, 4H); 1.99-2.02 (m, 4H). ESI MS (m/z): 366 (M+1)

Synthesis of Up109: 2-Oxo-7-azaspiro[3.5]nonane-7-carboxylate tert-butyl ester **1** (1.2 g, 1 eq) in Ethanol (20 mL) was added 30% Sodium Hydroxide aq.solution (4 eq,) at room temperature and 4-nitro benzaldehyde (1.51g, 2 eq) was added and stirred the reaction mixture at room temperature for 120 min. Water was added and the yellow precipitate was filtered, dried under vacuum and purified by column chromatography using hexanes: ethyl acetate (8:2). Removal of solvents under reduced pressure afforded **Up101** as yellow solid (2.04 g, 80.4%). 1HNMR (CDCl_3_, 400 MHz): 8.22-8.45 (m, 8H), 6.72 (s, 2H), 3.79-3.82 (m, 4H), 2.02-2.12(m, 4H), 1.67 (s, 9H). **Up101** (1.82 g) was dissolved in dioxane (10 mL) and treated with 4M HCl in dioxane (10 mL) and stirred at room temperature for 60 min. Solvents were removed under vacuum and the resultant yellow precipitate was washed with diethyl ether and dried under vacuum to afford **Up104** as yellow powder (0.772 g, 77.2%) ESI MS (m/z) 405.9. **Up104** (0.6 g, 1 eq) was dissolved in dichloromethane (15 mL) at 0^0^C and and triethyl amine (0.59 mL, 3 eq) was added and stirred for 5 min. To this mixture acryloyl chloride (108 µL,1.05 eq) was added dropwise and stirred the reaction mixture for 60 min. Reaction completion was monitored by TLC and 8% sodium bicarbonate aqueous solution was added and the organic layer was separated and washed with water, brine and dried over sodium sulfate. Removal of solvents under vacuum afforded yellow precipitate which was purified by column chromatography using hexanes:ethyl acetate (4:6) afforded **Up109** (0.494g, 78.5%) as an yellow powder. ^1^HNMR (CDCl_3_, 400 MHz): 8.14-8.48 (m, 8H); 6.67-6.89 (m, 3H); 6.33-6.41 (m, 1H); 5.78-5.81 (m, 1H); 3.79-4.09 (m, 4H); 2.01-2.12 (m, 4H). ESI MS (m/z): 459.2

Synthesis of Up148: To a stirred solution of 2-Oxo-7-azaspiro[3.5]nonane-7-carboxylate tert-butyl ester **1** (0.21 g, 1 eq) in Ethanol (10 mL) were added 30% Sodium Hydroxide aq.solution (4 eq,) and 2-thiazolecarboxaldehyde (0.374 g, 2 eq) at room temperature and stirring continued for 120 min. Water was added to the reaction mixture and the precipitated yellow solid was filtered, dried under vacuum and purified by column chromatography over silica gel using hexanes: ethyl acetate (8:2) as eluents. Removal of solvents under reduced pressure afforded **Up147** as yellow solid (0.28 g, 74.4%). To a stirred solutionjh of **Up147** (0.15 g) in dioxane (3 mL) was added 4M HCl in dioxane (3 mL) and stirring continued for 60 min at room temperature. Solvents were removed under vacuum and the resultant yellow precipitate was washed with diethyl ether and dried under vacuum to afford **Up148** as yellow powder (0.082 g, 65.3%) ESI MS (m/z) 330.

Synthesis of Up106: To a stirred solution of Boc-Phe-OH (0.172 g,1 eq) in DMF (5 mL) were added HBTU (0.246g, g, 1.1 eq), HOBt (0.085g, 1.1eq) and DIPEA (0.3 mL, 3seq) sequentially and stirring continued for 5 min. Up104 (0.26g, 1eq, in DMF) was added to the reaction mixture and stirring continued for overnight. Water was added to the reaction mixture and extracted with ethyl acetate two times. Organic layers were combined, washed with 10% sodium bicarbonate solution, saturated ammonium chloride, water and brine and dried over sodium sulfate. Removal of solvents under reduced pressure afforded Up105 as a crude compound which was purified by column chromatography over silica gel using hexanes: Ethyl acetate mixture (6:4) as eluents to give the title compound Up105 as a yellow solid (0.31 g 79.8%). To a stirred solution of Up105 (0.24 g) in dioxane (4 mL) was added 4M HCl in dioxane (4 mL) and stirring continued for 60 min. Solvents were removed under vacuum and the crude compound was precipitated in diethylether and dried under vacuum to give the title compound Up106 (0.17 g, 80.5%). ESIMS (m/z): 553

Synthesis of Up108 & Up112: To a stirred solution of 2-Oxo-7-azaspiro[3.5]nonane-7-carboxylate tert-butyl ester **1** (0.41 g, 1 eq) in Ethanol (10 mL) were added 30% Sodium Hydroxide aq.solution (4 eq,) and 3,4-dichlorobenzaldehyde (0.59 g, 2 eq) at room temperature and stirring continued for 120 min. Water was added to the reaction mixture and the precipitated yellow solid was filtered, dried under vacuum and purified by column chromatography over silica gel using hexanes: ethyl acetate (8:2) as eluents. Removal of solvents under reduced pressure afforded **Up107** as yellow solid (0.77g, 81.1%). ^1^HNMR (CDCl_3_, 400 MHz):δ 8.2 (s, 2H), 7.92-7.99 (d, 2H), 7.52-7.6(d, 2H), 6.51 (d, 2H), 3.69-3.72(m, 4H), 1.97-2.01(m, 4H), 1.69 (s, 9H).To a stirred solution of **Up107** (0.55 g) in dioxane (8 mL) was added 4M HCl in dioxane (8 mL) and stirring continued for 60 min at room temperature. Solvents were removed under vacuum and the resultant yellow precipitate was washed with diethyl ether and dried under vacuum to afford **Up108** as yellow powder (0.41g, 84.3%). ^1^HNMR (CD_3_OD, 400 MHz):δ 8.7 (s, 2H), 8.18-8.26 (d, 2H), 7.71-7.79 (d, 2H), 6.9 (s, 2H), 3.56-3.69 (m, 4H), 2.21-2.25 (m, 4H); ESI MS (m/z) 454 (M+1). To a stirred solution of Up108 (0.21 g, 1 eq) in dichloromethane (10 mL) was added di isopropyl ethylamine (0.22 mL, 3 eq) followed by acryloyl chloride (37 µL, 1.05 eq) at 0^0^C and stirred for 60 min at room temperature. Reaction completion was monitored by TLC. The reaction mixture was washed with saturated sodium bicarbonate aqueous solution (20mL), extracted the compound with dichloromethane, dried over sodium sulfate, filtered and concentrated. The crude product was purified by column chromatography over silica gel using hexanes: ethyl acetate (4:6) as eluents to give the title compound **Up112** (76%, 0.154 g) as an yellow powder (0.17g, 78.3%). ^1^HNMR (CDCl_3_, 400 MHz):δ 8.15 (s, 2H), 7.89-7.92 (d, 2H), 7.41-7.55 m(d, 2H), 6.55-6.61 (m, 1H), 6.46 (s, 2H), 6.27-6.31 (d, 1H), 5.68-5.72 (d, 1H), 3.71-3.9 (m, 4H), 1.88-1.98(m, 4H).

Synthesis of Up161:

Synthesis of Up200: To a stirred solution of Probenecid (0.142g, 1 eq) in DMF (3 mL) were added HBTU (0.2g, 1.1 eq), HOBt (0.074 g, 1.1eq) and DIPEA (253 µL, 3 eq) sequentially and stirring continued for 5 min. Up104 (0.22g, 1eq, in DMF) was added to the reaction mixture and stirring continued for overnight. Water was added to the reaction mixture and extracted with ethyl acetate two times. Organic layers were combined, washed with 10% sodium bicarbonate solution, saturated ammonium chloride, water and brine and dried over sodium sulfate. Removal of solvents under reduced pressure afforded Up200 as a crude compound which was purified by column chromatography over silica gel using hexanes: Ethyl acetate mixture (7:3) as eluents to give the title compound Up200 as a yellow solid (0.22 g, 65.6%). ESIMS (m/z): 690.9 (M+NH3)

Synthesis of Up173: To a stirred solution of 2-Oxo-6-azaspiro[3.4]octane-6-carboxylate tert-butyl ester **2** (0.27 g, 1 eq) in 20 mL Ethanol were added aq.30% Sodium Hydroxide solution (4 eq,) and 4-nitro benzaldehyde (0.362 g, 2 eq) at room temperature and stirring continued for 120 min. To the reaction mixture water was added and the precipitated yellow solid was filtered, dried under vacuum and purified by column chromatography over silica gel using hexanes: ethyl acetate (8:2) as eluents. Removal of solvents under reduced pressure afforded Up172 as yellow solid (0.43 g, 73.3 %). ^1^HNMR (CDCl_3_, 400 MHz): δ 8.23-8.41 (m, 8H); 6.62 (s, 2H); 3.66-3.79 (m, 4H); 2.3-2.39 (m, 2H), 1.52 (s, 9H) To a stirred solution of Up172 (0.36 g, 1 eq) in dioxane (5 mL) was added 4M HCl in dioxane (5 mL) and stirring continued for 60 min at room temperature. Solvents were removed under vacuum and the resultant yellow precipitate was precipitated with diethyl ether and dried under vacuum to afford **Up173** (0.28 g, 75.06 %) as yellow powder. ESI MS (m/z): 392 (M+1)

Synthesis of Up171: To a stirred solution of2-Oxo-6-azaspiro[3.4]octane-6-carboxylate tert-butyl ester **2** (0.16 g, 1 eq) in 5 mL Ethanol were added aq.30% Sodium Hydroxide solution (4 eq,) and 4-chloro benzaldehyde (0.199 g, 2 eq) at room temperature and stirring continued for 120 min. To the reaction mixture water was added and the precipitated light yellow solid was filtered, dried under vacuum and purified by column chromatography over silica gel using hexanes: ethyl acetate (8:2) as eluents. Removal of solvents under reduced pressure afforded Up168 as yellow solid (0.261 g, 78.1%). To a stirred solution of Up168 (0.22 g) in dioxane (4 mL) was added 4M HCl in dioxane (4 mL) and stirring continued for 60 min at room temperature. Solvents were removed under vacuum and the resultant yellow precipitate was precipitated with diethyl ether and dried under vacuum to afford **Up169** (0.116 g, 61%) as yellow powder. ESI MS (m/z): 370 (M+1). To a stirred solution of Up169 (81 mg, 1 eq) in DCM (5 mL) were added DIPEA (98 µL, 3 eq) and Acryloyl chloride (17 µL, 1 eq) at 0^0^C and stirring continued for 60 min at room temperature. Reaction completion was monitored by TLC. The reaction mixture was washed with saturated sodium bicarbonate aqueous solution (20mL), extracted the compound with dichloromethane, dried over sodium sulfate, filtered and concentrated. The crude product was purified by column chromatography over silica gel using hexanes:ethyl acetate (4:6) as eluents to give the title compound **Up171** (66.1 %, 56.1 mg) as an yellow powder. ESI MS (m/z): 424 (M+1)

Synthesis of Up302 and Up306: To a stirred solution of 2-Oxo-6-azaspiro[3.4]octane-6-carboxylate tert-butyl ester **2** (1.008, 1 eq) in 20 mL Ethanol were added aq.30% Sodium Hydroxide solution (4 eq, 2.4 mL) and 4-cyano benzaldehyde (1.18 g, 2 eq) at room temperature and stirring continued for 120 min. To the reaction mixture water was added and the precipitated light yellow solid was filtered, dried under vacuum and purified by column chromatography over silica gel using hexanes: ethyl acetate (8:2) as eluents. Removal of solvents under reduced pressure afforded Up301 as yellow solid (1.6 g, 82.4%). To a stirred solution of Up301 (1.2 g) in dioxane (10 mL) was added 4M HCl in dioxane (10 mL) and stirring continued for 60 min at room temperature. Solvents were removed under vacuum and the resultant yellow precipitate was precipitated with diethyl ether and dried under vacuum to afford **Up302** (0.78 g, 75.7%) as yellow powder. ESI MS (m/z): 352 (M+1). To a stirred solution of Up302 (0.21 g, 1 eq) in dichloromethane (10 mL) was added di isopropyl ethylamine (0.22 mL, 3 eq) followed by acryloyl chloride (37 µL, 1.05 eq) at 0^0^C and stirred for 60 min at room temperature. Reaction completion was monitored by TLC. The reaction mixture was washed with saturated sodium bicarbonate aqueous solution (20mL), extracted the compound with dichloromethane, dried over sodium sulfate, filtered and concentrated. The crude product was purified by column chromatography over silica gel using hexanes: ethyl acetate (4:6) as eluents to give the title compound **Up306** (76%, 0.154 g) as an yellow powder (0.17g, 78.3%).

Synthesis of Up188: To a stirred solution of Up104 (76 mg, 1 eq) in DCM (5 mL) were added DIPEA (90 µL, 3 eq) and benzenesulfonyll chloride (22 µL, 1 eq) at 0^0^C and stirring continued for 60 min at room temperature. Reaction completion was monitored by TLC. The reaction mixture was washed with saturated sodium bicarbonate aqueous solution (10mL), extracted the compound with dichloromethane, dried over sodium sulfate, filtered and concentrated. The crude product was purified by column chromatography over silica gel using hexanes:ethyl acetate (4:6) as eluents to give the title compound **Up188** (71.2%, 67 mg) as an yellow powder. ESI MS (m/z): 546 (M+1)

Synthesis of Up288: To a stirred solution of Up284 (56.6 mg,1 eq) in DCM (5 mL) were added DIPEA (75 µL, 3 eq) and benzoyl chloride (22.2 µL,1 eq) at 0^0^C and stirring continued for 60 min at room temperature. Reaction completion was monitored by TLC. The reaction mixture was washed with saturated sodium bicarbonate aqueous solution (10mL), extracted the compound with dichloromethane, dried over sodium sulfate, filtered and concentrated. The crude product was purified by column chromatography over silica gel using hexanes:ethyl acetate (1:1) as eluents to give the title compound **Up288** (61.5%, 42 mg) as an yellow powder. ESI MS (m/z): 470 (M+1)

Synthesis of Up290: To a stirred solution of Up284 (53.2 mg, 1 eq) in DCM (5 mL) were added DIPEA (72µL, 3 eq) and Trifluoroacetic anhydride (19 µL,1 eq) at 0^0^C and stirring continued for 60 min at room temperature. Reaction completion was monitored by TLC. The reaction mixture was washed with saturated sodium bicarbonate aqueous solution (20mL), extracted the compound with dichloromethane, dried over sodium sulfate, filtered and concentrated. The crude product was purified by column chromatography over silica gel using hexanes:ethyl acetate (7:3) as eluents to give the title compound **Up290** (74.6%, 47 mg) as an yellow powder. ESI MS (m/z): 462 (M+1)

Synthesis of Up199 and Up201: To a stirred solution of 2-Oxo-7-azaspiro[3.5]nonane-7-carboxylate tert-butyl ester **1** (0.24 g, 1 eq) in Ethanol (10 mL) were added 30% Sodium Hydroxide aq.solution (4 eq,) and 4-bromobenzaldehyde (0.371 g, 2 eq) at room temperature and stirring continued for 120 min. Water was added to the reaction mixture and the precipitated yellow solid was filtered, dried under vacuum and purified by column chromatography over silica gel using hexanes: ethyl acetate (8:2) as eluents. Removal of solvents under reduced pressure afforded **Up197** as yellow solid (0.484 g, 85.3 %). To a stirred solution of **Up197** (0.4 g) in dioxane (6 mL) was added 4M HCl in dioxane (6 mL) and stirring continued for 60 min at room temperature. Solvents were removed under vacuum and the resultant yellow precipitate was washed with diethyl ether and dried under vacuum to afford **Up198** as yellow powder (0.29 g, 81.9%) ESI MS (m/z) 472. To a stirred solution of Up198 (0.11 g, 1 eq) in dichloromethane (6 mL) was added diisopropyl ethylamine (0.12 mL, 3 eq) followed by acryloyl chloride (19 µL, 1.05 eq) at 0^0^C and stirred for 60 min at room temperature. Reaction completion was monitored by TLC. The reaction mixture was washed with saturated sodium bicarbonate aqueous solution (20mL), extracted the compound with dichloromethane, dried over sodium sulfate, filtered and concentrated. The crude product was purified by column chromatography over silica gel using hexanes: ethyl acetate (4:6) as eluents to give the title compound **Up199** as an yellow powder (0.088g, 77.1%) ESIMS (m/z: 528-M+1). To a stirred solution of Up198 in DMF (mL) were added K_2_CO_3_ and 1-bromomethylnaphthalene at room temperature and stirring continued for overnight at 90^0^ C. Water was added to the reaction mixture and extracted with ethyl acetate, washed with brine, dried over sodium sulfate and concentrated under vacuum. The crude compound was purified by column chromatography over silica gel using hexanes:ethylacetate (8:2) to give the title compound Up201 as yellow solid. ESI MS (m/z: 631-M+H2O).

Synthesis of Up142: To a stirred solution of 2-Oxo-7-azaspiro[3.5]nonane-7-carboxylate tert-butyl ester **1** (0.17 g, 1 eq) in Ethanol (10 mL) were added 30% Sodium Hydroxide aq.solution (4 eq,) and benzaldehyde (0.263 g, 2 eq) at room temperature and stirring continued for 120 min. Water was added to the reaction mixture and the precipitated yellow solid was filtered, dried under vacuum and purified by column chromatography over silica gel using hexanes: ethyl acetate (8:2) as eluents. Removal of solvents under reduced pressure afforded **Up138** as yellow solid (0.36 g, 78.9 %). To a stirred solution of **Up138** (0.3 g) in dioxane (6 mL) was added 4M HCl in dioxane (6 mL) and stirring continued for 60 min at room temperature. Solvents were removed under vacuum and the resultant yellow precipitate was washed with diethyl ether and dried under vacuum to afford **Up140**as yellow powder (0.18 g,71.1%) ESI MS (m/z) 316. To a stirred solution of Up140 (0.11 g, 1 eq) in dichloromethane (6 mL) was added diisopropyl ethylamine (0.16 mL, 3 eq) followed by acryloyl chloride (24 µL, 1.05 eq) at 0^0^C and stirred for 60 min at room temperature. Reaction completion was monitored by TLC. The reaction mixture was washed with saturated sodium bicarbonate aqueous solution (20mL), extracted the compound with dichloromethane, dried over sodium sulfate, filtered and concentrated. The crude product was purified by column chromatography over silica gel using hexanes: ethyl acetate (4:6) as eluents to give the title compound **Up142** as an yellow powder (0.08g, 68.9%) ESIMS (m/z: 370-M+1).

Synthesis of Up132: To a stirred solution of 2-Oxo-7-azaspiro[3.5]nonane-7-carboxylate tert-butyl ester **1** (0.2 g, 1 eq) in Ethanol (10 mL) were added 30% Sodium Hydroxide aq.solution (4 eq,) and 4-fluoro benzaldehyde (0.207 g, 2 eq) at room temperature and stirring continued for 120 min. Water was added to the reaction mixture and the precipitated yellow solid was filtered, dried under vacuum and purified by column chromatography over silica gel using hexanes: ethyl acetate (8:2) as eluents. Removal of solvents under reduced pressure afforded **Up130** as yellow solid (0.26 g, 76.9 %). To a stirred solution of **Up130** (0.22 g) in dioxane (6 mL) was added 4M HCl in dioxane (6 mL) and stirring continued for 60 min at room temperature. Solvents were removed under vacuum and the resultant yellow precipitate was washed with diethyl ether and dried under vacuum to afford **Up131** as yellow powder (0.137 g, 72.8 %) ESI MS (m/z) 353. To a stirred solution of Up131 (0.086 g, 1 eq) in dichloromethane (4 mL) was added diisopropyl ethylamine (0.12 mL, 3 eq) followed by acryloyl chloride (18 µL, 1.05 eq) at 0^0^C and stirred for 60 min at room temperature. Reaction completion was monitored by TLC. The reaction mixture was washed with saturated sodium bicarbonate aqueous solution (12 mL), extracted the compound with dichloromethane, dried over sodium sulfate, filtered and concentrated. The crude product was purified by column chromatography over silica gel using hexanes: ethyl acetate (4:6) as eluents to give the title compound **Up132** as an yellow powder (0.062 g, 68.8%) ESIMS (m/z: 406-M+1)

Synthesis of Up135: To a stirred solution of 2-Oxo-7-azaspiro[3.5]nonane-7-carboxylate tert-butyl ester **1** (0.24 g, 1 eq) in Ethanol (10 mL) were added 30% Sodium Hydroxide aq.solution (4 eq,) and 2-fluoro benzaldehyde (0.249 g, 2 eq) at room temperature and stirring continued for 120 min. Water was added to the reaction mixture and the precipitated yellow solid was filtered, dried under vacuum and purified by column chromatography over silica gel using hexanes: ethyl acetate (8:2) as eluents. Removal of solvents under reduced pressure afforded **Up133** as yellow solid (0.347 g, 83.4 %). To a stirred solution of **Up133** (0.29 g) in dioxane (6 mL) was added 4M HCl in dioxane (6 mL) and stirring continued for 60 min at room temperature. Solvents were removed under vacuum and the resultant yellow precipitate was washed with diethyl ether and dried under vacuum to afford **Up134** as yellow powder (0.183 g, 75 %) ESI MS (m/z) 353. To a stirred solution of Up134 (0.124 g, 1 eq) in dichloromethane (4 mL) was added diisopropyl ethylamine (0.16 mL, 3 eq) followed by acryloyl chloride (25 µL, 1.05 eq) at 0^0^C and stirred for 60 min at room temperature. Reaction completion was monitored by TLC. The reaction mixture was washed with saturated sodium bicarbonate aqueous solution (12 mL), extracted the compound with dichloromethane, dried over sodium sulfate, filtered and concentrated. The crude product was purified by column chromatography over silica gel using hexanes: ethyl acetate (4:6) as eluents to give the title compound **Up135** as an yellow powder (0.087 g, 67.4%) ESIMS (m/z: 406-M+1)

Synthesis of Up137: To a stirred solution of 2-Oxo-7-azaspiro[3.5]nonane-7-carboxylate tert-butyl ester **1** (0.12 g, 1 eq) in Ethanol (10 mL) were added 30% Sodium Hydroxide aq.solution (4 eq,) and cinnamaldehyde (0.132 g, 2 eq) at room temperature and stirring continued for 120 min. Water was added to the reaction mixture and the precipitated yellow solid was filtered, dried under vacuum and purified by column chromatography over silica gel using hexanes: ethyl acetate (8:2) as eluents. Removal of solvents under reduced pressure afforded **Up136** as yellow solid (0.17 g, 72.64 %). To a stirred solution of **Up136** (0.15 g) in dioxane (4 mL) was added 4M HCl in dioxane (4 mL) and stirring continued for 60 min at room temperature. Solvents were removed under vacuum and the resultant yellow precipitate was washed with diethyl ether and dried under vacuum to afford **Up137** as yellow powder (0.09 g, 69.7 %) ESI MS (m/z) 368.

Synthesis of Up144: To a stirred solution of 2-Oxo-7-azaspiro[3.5]nonane-7-carboxylate tert-butyl ester **1** (0.18 g, 1 eq) in Ethanol (10 mL) were added 30% Sodium Hydroxide aq.solution (4 eq,) and picolinaldehyde (0.161 g, 2 eq) at room temperature and stirring continued for 120 min. Water was added to the reaction mixture and the precipitated yellow solid was filtered, dried under vacuum and purified by column chromatography over silica gel using hexanes: ethyl acetate (7:3) as eluents. Removal of solvents under reduced pressure afforded **Up143** as yellow solid (0.23 g, 73.2 %). To a stirred solution of **Up143** (0.2 g) in dioxane (4 mL) was added 6M HCl in dioxane (6 mL) and stirring continued for 60 min at room temperature. Solvents were removed under vacuum and the resultant yellow precipitate was washed with diethyl ether and dried under vacuum to afford **Up144** as yellow powder (0.18 g, 73.6 %) ESI MS (m/z) 318.

Synthesis of Up291: To a stirred solution of valproic acid (0.056 g, 1.1 eq) in DMF (5 mL) were added HBTU (0.146 g, 1.1 eq), HOBt (0.05 g, 1.1eq) and DIPEA (180 µL, 3 eq) sequentially and stirring continued for 5 min. Up284 (0.14 g, 1eq, in DMF) was added to the reaction mixture and stirring continued for overnight. Water was added to the reaction mixture and extracted with ethyl acetate two times. Organic layers were combined, washed with 10% sodium bicarbonate solution, saturated ammonium chloride, water and brine and dried over sodium sulfate. Removal of solvents under reduced pressure afforded Up291 as a crude compound which was purified by column chromatography over silica gel using hexanes: Ethyl acetate mixture (6:4) as eluents to give the title compound Up291 as a yellow solid (0.13 g, 76%). ESIMS (m/z): 492 (M+1)

Synthesis of Up292: To a stirred solution of dimethylglycine (0.04 g, 1.1 eq) in DMF (5 mL) were added HBTU (0.146 g, 1.1 eq), HOBt (0.05 g, 1.1eq) and DIPEA (180 µL, 3 eq) sequentially and stirring continued for 5 min. Up284 (0.14 g, 1eq, in DMF) was added to the reaction mixture and stirring continued for overnight. Water was added to the reaction mixture and extracted with ethyl acetate two times. Organic layers were combined, washed with 10% sodium bicarbonate solution, saturated ammonium chloride, water and brine and dried over sodium sulfate. Removal of solvents under reduced pressure afforded Up292 as a crude compound which was purified by column chromatography over silica gel using hexanes: Ethyl acetate mixture (6:4) as eluents to give the title compound Up292 as a yellow solid (0.121 g, 76.5%). ESIMS (m/z): 451 (M+1)
